# Inhibition of Astrocytic JMJD3 Attenuates Neuroinflammation-Mediated Blood–Brain Barrier Disruption and Improves Functional Recovery After Intracerebral Hemorrhage in Mice

**DOI:** 10.3390/brainsci16050454

**Published:** 2026-04-24

**Authors:** Quan Zhang, Dewen Ru, Jiang Fang, Jun Zeng, Qiang Yuan, Zhuoying Du, Gang Wu, Jianhong Zhu, Jin Hu

**Affiliations:** 1 Department of Neurosurgery, Huashan Hospital of Fudan University, Shanghai 200040, China; 19111220079@fudan.edu.cn (Q.Z.); 13211270010@fudan.edu.cn (D.R.); 19111220082@fudan.edu.cn (J.F.); jzhu@fudan.edu.cn (J.Z.); 2 Department of Neurosurgery, Jinshan Hospital of Fudan University, Shanghai 201508, China; 3 Department of Neurosurgery, The First Affiliated Hospital of Chongqing Medical University, Chongqing 400016, China

**Keywords:** intracerebral hemorrhage, JMJD3, astrocytes, neuroinflammation, blood–brain barrier, MMP-9, histone demethylase, GSK-J4

## Abstract

**Highlights:**

**What are the main findings?**

- Histone demethylase JMJD3 is highly upregulated in murine astrocytes after intracerebral hemorrhage (ICH) in vitro and in vivo and correlates with neuroinflammation.
- JMJD3 inhibition by GSK-J4 attenuates astrocytic neuroinflammation and preserves blood–brain barrier integrity in acute mouse ICH model, likely via MMP-9 suppression.

**What are the implications of the main findings?**

- Astrocytic JMJD3 is a key regulator of neuroinflammation after ICH and represents a potential therapeutic target in the acute phase.
- GSK-J4 confers histological and functional neuroprotection in acute ICH mice, highlighting its therapeutic potential.

**Abstract:**

Background: Intracerebral hemorrhage (ICH) is a devastating subtype of stroke, in which neuroinflammation and blood–brain barrier (BBB) disruption are secondary pathophysiological events that drive progressive brain injury. Histone lysine demethylase JMJD3 (Jumonji C domain-containing protein 3) is a master epigenetic switch governing inflammatory signaling; however, its participation in ICH-induced vascular disruption and its possible mechanism remain elusive. Objective: To examine the expression patterns of JMJD3 in the context of ICH and to evaluate the therapeutic potential of its specific inhibitor, GSK-J4, in attenuating neuroinflammation and BBB disruption in a murine ICH model. Methods: Hemin treatment of a mouse C8-D1A astrocytic cell line was used to develop an in vitro ICH model. The transcript level of the *Jmjd3* gene and its correlation with pro-inflammatory signaling were analyzed with or without GSK-J4 pretreatment. ICH in vivo was created experimentally in adult male C57BL/6 mice through stereotactic striatal injection of collagenase IV, and the mice were randomly assigned to sham, ICH + vehicle, and ICH + GSK-J4 (30 mg/kg intraperitoneally (i.p.), every other day starting three days before ICH) groups. At three days post-ICH, ipsilateral brain tissues were collected to detect JMJD3 cellular localization, pro-inflammatory mediator levels, tight junction protein expression, BBB ultrastructure, and hematoma volume. White matter integrity and neuronal recovery were assessed on day 7, and sensorimotor function was assessed longitudinally on days 1, 3, 5, 7, and 14. Results: *Jmjd3* gene transcription was upregulated in hemin-treated astrocytes and correlated positively with IL-6 pro-inflammatory signaling activation. In vivo, the co-localization of JMJD3 with the astrocytic identifier glial fibrillary acidic protein (GFAP) was markedly increased in the area adjacent to the hematoma at three days post-ICH. GSK-J4 administration significantly suppressed the pro-inflammatory signaling cascade by decreasing the levels of inducible nitric oxide synthase (iNOS), tumor necrosis factor-α (TNF-α), and matrix metalloproteinase-9 (MMP-9), enhanced brain vascular structural and functional integrity by upregulating tight junction proteins zonula occludens protein-1 (ZO-1) and claudin-5, improved BBB ultrastructural integrity, and decreased hematoma volume at three days post-ICH. Furthermore, GSK-J4 administration promoted white matter integrity (increased myelin basic protein [MBP] expression) and neuronal recovery (increased neuron-specific nuclear protein [NeuN] expression) at seven days post-ICH and significantly improved the performance of ICH mice in sensorimotor behavioral tests. Conclusions: Astrocytic JMJD3 is upregulated following ICH and promotes neuroinflammation, which in turn mediates BBB disruption. Pharmacological inhibition of JMJD3 by GSK-J4 attenuates neuroinflammation and subsequent BBB damage, accelerates hematoma resolution, and promotes histological and functional recovery after ICH, likely by downregulating MMP-9 expression. These findings identify astrocytic JMJD3 as a novel epigenetic therapeutic target for acute ICH.

## 1. Introduction

Spontaneous intracerebral hemorrhage (ICH) constitutes 27% of all incident strokes worldwide, imposing a significant socioeconomic and clinical disease burden [1]. Pathologically, ICH is characterized by acute hematoma formation in the brain parenchyma, followed by rapid red blood cell (RBC) degradation and the release of multiple cytotoxic byproducts. ICH-induced brain injury is a biphasic process: primary injury arises from direct hematoma compression of neural and vascular tissues, whereas secondary injury is driven by delayed toxic effects of RBC lysis products, particularly hemin, iron, carbon monoxide, and bilirubin [2,3]. Hemin is a key mediator that triggers excessive reactive oxygen species (ROS) and peroxynitrite formation, which directly induces blood–brain barrier (BBB) disruption [4]. BBB disruption not only contributes to acute neurological deficits but also accelerates delayed brain damage after ICH. As a central pathological event, BBB disruption represents a promising therapeutic target for neuroprotective intervention [5,6,7].

Neuroinflammation is another core mechanism underlying ICH-induced delayed brain damage, initiated via the cross-linked activation of resident central nervous system (CNS) neuroglial cells, primarily astrocytes and microglia [8,9]. Moderate glial activation contributes to cell debris clearance and preservation of healthy tissue; however, during the early phase of ICH, damage-associated molecular patterns (DAMPs) originating from the lesioned cerebral parenchyma drive excessive glial activation toward a pro-inflammatory phenotype [8,9], recruiting peripheral leukocytes to the injured site by releasing inflammatory signaling molecules [10]. The robust reactivity of neuroglia and other circulating immune cells initiates an inflammatory cascade that attacks brain tissue and exacerbates brain damage [11,12].

Astrocytes are the most prevalent neuroglial population and function in preserving brain homeostasis, providing architectural and nutritional support to neurons, and regulating synaptic activity [13]. Notably, perivascular astrocytic endfeet are integral components of the BBB. They encircle the entire cerebrovascular tree and are strategically located to support neurovascular coupling, substance exchange, and vascular tone regulation [14,15]. Accumulating evidence has identified astrocyte-mediated neuroinflammation and neurotoxicity as key initiators of ICH-induced BBB disruption and neuronal cell loss [16,17]. Mechanistically, during the acute period following ICH, thrombin, a major DAMP released from the hematoma, can bind to protease-activated receptors (PARs) on perisynaptic astrocytic endfeet. PARs are thrombin receptors that activate Src kinase signaling, which can exert profound influence on downstream inflammatory cascades and ultimately induce apoptotic neuronal death and endothelial damage [18,19]. Furthermore, reactive astrocytes are stimulated by microglia-derived inflammatory signal molecules (interleukin-1β [IL-1β], tumor necrosis factor-α [TNF-α]) and complement components (e.g., complement 1q [C1q]), which in turn secrete cytokines interleukin-15 (IL-15), chemokines (C-C motif chemokine ligand 2 [CCL2], C-C motif chemokine ligand 5 [CCL5]), and damage agents, including ROS, nitric oxide (NO), and the matrix metalloproteinase-9 (MMP-9) [20,21]. These factors not only mediate intercellular crosstalk with recruited immune cells to amplify inflammation but also directly degrade BBB structural components, leading to BBB disruption and neuronal injury [22,23]. Collectively, these findings emphasize the potential of modulating astrocyte-mediated CNS inflammation and BBB disruption in acute ICH treatment.

Histone methylation is a pivotal epigenetic modification of gene transcription promoted by histone lysine methyltransferases (KMTs). However, this modification is dynamic, as its removal can be actively mediated by histone lysine demethylases (KDMs), which exert opposing functions [24]. As epigenetic “erasers”, KDMs catalyze the elimination of methyl groups within histone H3 and H4 lysine positions, and their dysregulation has been implicated in developmental defects, cancer, inflammatory diseases, and neurodegenerative disorders [25]. Mounting evidence has demonstrated that the downregulation of specific KDM families (e.g., KDM1 and KDM4) contributes to neuroprotective effects in ischemic stroke, experimental autoimmune encephalomyelitis, and traumatic brain injury (TBI) [26,27,28,29,30]. However, most well-characterized KDM families exhibit dual substrate specificity: KDM4 demethylates both repressive H3K9me2/3 and activating H3K36me2/3, whereas KDM1 demethylates activating H3K4me1/2 and repressive H3K9me1/2 [31,32]. Since demethylation of H3K4/H3K36 inhibits transcription while demethylation of H3K9/H3K27 activates it, non-selective inhibition of KDMs can cause off-target effects. Consequently, these off-target effects impede the clinical development of their inhibitors for neuroinflammation [33]. Therefore, identifying substrate-specific KDMs that drive ICH-induced neuroinflammation is critical for developing targeted epigenetic therapies for ICH.

Jumonji C domain-containing protein 3 (JMJD3/KDM6B), belonging to the KDM6 family, displays high substrate specificity and mediates the erasure of the gene-silencing histone mark, histone H3 at lysine(K) 27 trimethylation (H3K27me3), thus facilitating the transcription of various inflammatory genes [34]. Unlike other KDMs, H3K27me3 demethylation by JMJD3 is a highly reversible process, and its pro-inflammatory role has been well documented in various systemic and CNS inflammatory diseases [33,34]. In the CNS, JMJD3-mediated neuroinflammation has been implicated in ischemic stroke, spinal cord injury (SCI), and neuropsychiatric disorders [35,36,37,38]. In vitro studies have shown elevated JMJD3 expression in ischemic astrocytes and in mechanically injured astroglial cell models, indicating that increased JMJD3 levels are correlated with astrocytic apoptosis and neuroinflammation [39,40]. A recent study reported that in vivo JMJD3 inhibition attenuates neuroinflammation and BBB impairment in a murine TBI model, potentially by targeting brain endothelial cells [41]. However, the involvement of astrocyte-specific JMJD3 in post-ICH CNS inflammation, vascular dysfunction, and subsequent BBB disruption remains unclear, representing a critical knowledge gap in the epigenetic regulation of acute ICH.

GSK-J4 is a lipophilic, brain-penetrant, and selective inhibitor of JMJD3 (and UTX/KDM6A). As a prodrug of GSK-J1, GSK-J4 contains an ethyl ester moiety that enhances its membrane permeability. Upon cellular entry, GSK-J4 is hydrolyzed to GSK-J1, which competitively inhibits JMJD3 enzymatic activity [33]. GSK-J4 has emerged as a valuable pharmacological tool for investigating JMJD3 function in CNS pathologies, with proven neuroprotective effects in models of lipopolysaccharide (LPS)-induced anxiety and spinal cord injury [36,37]. Given the pivotal function of astrocytes in ICH-induced neuroinflammation and BBB disruption, we hypothesized that JMJD3 is upregulated in reactive astrocytes following ICH and drives neuroinflammation-mediated BBB disruption and its pharmacological inhibition by GSK-J4 attenuates these pathological processes, accelerates hematoma resolution, and improves neurological performance of mice in acute ICH.

In this study, we generated an in vitro ICH model using hemin-treated mouse C8-D1A astrocytes and an in vivo collagenase-induced mouse ICH model to: (1) characterize the expression pattern of JMJD3 in astrocytes post-ICH; (2) verify the pro-inflammatory role of astrocytic JMJD3 in ICH-induced BBB disruption; (3) evaluate the therapeutic effects of GSK-J4 on neuroinflammation, BBB integrity, hematoma resolution, and sensorimotor function recovery in acute ICH; and (4) explore the underlying mechanism involving MMP-9-mediated BBB structural damage. Our findings identify astrocytic JMJD3 as a novel substrate-specific epigenetic therapeutic target for acute ICH, providing a new mechanistic basis for developing targeted anti-inflammatory therapies for this devastating stroke subtype.

## 2. Materials and Methods

### 2.1. Drugs and Reagents

GSK-J4 (Catalogue Number: HY-15648B, molecular weight: 417.50, formula: C24H27N5O2, purity: ≥99%) was purchased from MedChemExpress (Shanghai, China). For in vivo administration, GSK-J4 was firstly dissolved within dimethyl sulfoxide (DMSO; Sigma-Aldrich, St. Louis, MO, USA) to prepare a concentrated mother solution. This mother solution was further diluted with corn oil (Catalogue Number: ST1177, Beyotime Biotechnology, Haimen, China) to achieve a final vehicle composition of 10% DMSO/90% corn oil (*v*/*v*). For in vitro use, GSK-J4 was solubilized in DMSO and then diluted 1:1000 with saline to a final DMSO concentration of 0.1% (*v*/*v*).

To establish ICH models, collagenase type IV (Catalogue Number: C4-BIOC; Sigma-Aldrich, St. Louis, MO, USA) was dissolved in saline for in vivo procedures, whereas hemin (Catalogue Number: H9039, Sigma-Aldrich, St. Louis, MO, USA) was solubilized in 0.1% DMSO for the in vitro experiments. An interleukin-6 enzyme-linked immunosorbent assay (ELISA) kit to quantify interleukin-6 (IL-6) in astrocyte culture supernatants was obtained from Elabscience Biotechnology (Wuhan, China). The following antibodies were used: rabbit polyclonal antibodies against JMJD3 (Catalogue Number: NBP1-06640), goat polyclonal antibody against platelet endothelial cell adhesion molecule 1 (CD31) (Catalogue Number: AF3628), rabbit polyclonal antibodies against claudin-5 (Catalogue Number: NBP2-92846) from Novus Biologicals (Centennial, CO, USA); rat monoclonal antibody against neuron-specific nuclear protein (NeuN) (Catalogue Number: ab279297), rat monoclonal antibody against glial fibrillary acidic protein (GFAP) (Catalogue Number: ab279291), rat monoclonal antibody against ionized calcium binding adaptor molecule-1 (Iba1) (Catalogue Number: ab283346), rabbit monoclonal antibody against CD68 (Catalogue Number: ab283654), rabbit monoclonal antibody against zonula occludens protein-1 (ZO-1) (Catalogue Number: ab276131), rabbit monoclonal antibody against TNF-α (Catalogue Number: ab215188) from Abcam (Cambridge, UK); rabbit monoclonal antibody against myelin basic protein (MBP) (Catalogue Number: 78896) from Cell Signaling Technology (Danvers, MA, USA); rabbit polyclonal antibody against inducible nitric oxide synthase (iNOS) (Catalogue Number: 22226-1-AP), rabbit polyclonal antibody against MMP-9 (Catalogue Number: 10375-2-AP) from Proteintech (Wuhan, China); rabbit monoclonal antibody against beta-actin (β-actin) (Catalogue Number: AC038) from Abclonal Technology (Wuhan, China). The dilutions used for each antibody are detailed in the corresponding Methods sections.

### 2.2. Cell Culture and In Vitro Intervention

For in vitro experiments, the mouse astrocyte type I clone cell line (C8-D1A) [42] was obtained commercially from the FuHeng Cell Center (Catalogue Number: FH0837; Shanghai, China). Cells were cultured as adherent monolayers in complete DMEM/F12 medium (Gibco, New York, NY, USA) at 37 °C in a temperature-controlled incubator equipped with a water pan to maintain humidity and supplied with 5% CO_2_. The culture medium contained 10% fetal bovine serum (FBS; Gibco) and 1% penicillin G-streptomycinum (Gibco). Confluent (80–90%) C8-D1A cells were detached with 0.25% trypsin (Nacalai Tesque, Kyoto, Japan) at 37 °C for 5 min. After resuspension, cells were seeded into 24-well and 96-well plates at densities of 2.5 × 10^5^ and 1 × 10^4^ cells per well. Following plating, cells were maintained for 24 h to permit adhesion prior to exposure to hemin for in vitro injury induction.

The in vitro experiment consisted of the following groups: (1) Control group: C8-D1A cells were cultured with 0.1% DMSO as a vehicle for 12 h. (2) Hemin group: C8-D1A cells were incubated with the ICH-inducing agent hemin (30 μM in 0.1% DMSO) for 12 h, and for time-dependent analysis, cells were obtained at predetermined time intervals (6, 12, and 48 h). (3) Hemin + GSK-J4 group: C8-D1A cells were pre-incubated with 5 μM GSK-J4 for 30 min prior to the administration of hemin. The in vitro doses of hemin and GSK-J4 were selected based on previous studies [43,44]. After 12 h of incubation, the cells harvested from 24-well plates and the supernatants harvested from 96-well plates were used for RNA extraction and ELISA, respectively. Each experimental group was analyzed in three independent biological replicates.

### 2.3. RNA Sequencing and Bioinformatic Analysis

In accordance with previous protocols [44], transcriptomic profiling and RNA computational analysis were performed on C8-D1A astrocytes in the hemin group and control group, incorporating three biological replicates per group. In brief, 1 mL of TRIzol (Catalogue Number: 15596026; Thermo Fisher Scientific, Waltham, MA, USA) was added to each well of a 6-well plate for total RNA purification from one replicate per group. RNA extracts were measured using a NanoDrop 2000 spectrophotometer (Thermo Fisher). The RNA integrity number (RIN) of the extracts was determined using an Agilent 2100 Bioanalyzer (Agilent Technologies, Santa Clara, CA, USA) with the RNA 6000 Nano Kit (Catalogue Number: 5067-1511, Agilent Technologies), and RIN values exceeding 7.0 being considered acceptable. Subsequently, using the NEBNext^®^ Ultra RNA Library Prep Kit (NEB, Ipswich, MA, USA), sequencing libraries were constructed. These libraries were subjected to paired-end sequencing (2 × 150 bp reads) on a HiSeq X Ten platform (Illumina, San Diego, CA, USA).

For RNA bioinformatic analysis, Trimmomatic (version 0.39) was first employed to eliminate adapter sequences and filter out low-quality reads from the raw sequencing data. Following this preprocessing step, HISAT2 software (version 2.2.1) was used to align the filtered reads to the mm10 reference genome assembly. Subsequently, HTSeq-count software (version 2.0.2) was employed to quantify gene expression and derive raw counts for the identified genes. Differential expression analysis was then performed using the DESeq2 R package (version 1.46.0) based on raw counts data. Genes with a fold change >1.5 or <0.67 and an adjusted *p*-value < 0.05 were considered significantly differentially expressed genes (DEGs). Furthermore, we performed Gene Ontology (GO) enrichment analysis to explore the functional roles of the identified genes using the clusterProfiler R package (version 4.18.1), and the analysis was focused on molecular function (MF) to show the most enriched terms. In parallel, gene set enrichment analysis (GSEA) was conducted to evaluate the enrichment of the MF term “histone demethylase activity” based on a pre-ranked gene list, with enrichment quantified by the normalized enrichment score (NES) and statistical significance defined as a false discovery rate (FDR) < 0.25. For the correlation analysis, the gene counts underwent a transformation to FPKM (fragments per kilobase per million) using standard normalization methods. The relationship between *Jmjd3* expression (log2(FPKM+1)) and the activity of pro-inflammatory hallmark pathways (obtained through the Molecular Signatures Database, MSigDB) was evaluated by Pearson’s correlation, following the quantification of gene set variation analysis (GSVA) using the GSVA R package (version 2.4.8). These procedures provided insights into the interplay between gene expression and functional pathways in the biological context being studied.

### 2.4. Quantitative Real-Time Polymerase Chain Reaction (RT-qPCR)

RT-qPCR analysis was performed using samples from the hemin and control groups. First, total RNA was extracted from fully confluent astrocytes grown in each well of a 24-well plate using 200 μL of TRIzol. The RNA concentrations were then adjusted to contain equal amounts (800 ng per sample) for reverse transcription of complementary DNA (cDNA) after quantification using NanoDrop 2000. In this study, cDNA synthesis was executed by the PrimeScript cDNA synthesis kit (TaKaRa, Shiga, Japan). PCR was then performed with each reaction set up in a total volume of 20 μL. The contents of the reaction mixture for one sample contained 2 μL of cDNA, 0.8 μL of both forward and reverse primers (each at 10 μM), 10 μL of the 2× SYBR Green Master Mix from TaKaRa, and 6.4 μL of RNase-free water. For accuracy, the cycle threshold (Ct) values were generated from the average of three repeats per sample. Thermal cycling was conducted using a StepOnePlus thermal cycler (Applied Biosystems, Foster City, CA, USA) following the supplier’s protocol. The mRNA levels of selected genes were quantified using the 2^−ΔΔCt^ method. Briefly, the threshold cycle (Ct) for each gene was determined, and the ΔCt was calculated by subtracting the Ct of the reference gene glyceraldehyde-3-phosphate dehydrogenase (*Gapdh*) from the Ct of the target gene. The ΔΔCt was then obtained by subtracting the average ΔCt of the control group from the ΔCt of each experimental sample. Finally, the relative expression level was determined as 2^−ΔΔCt^. Each group was analyzed in three biological replicates. The sequences and properties of the primers are listed in Table 1.

### 2.5. Enzyme-Linked Immunosorbent Assay (ELISA) Detection

After 12 h of incubation, the supernatants were harvested from each sample from the control, hemin, and hemin + GSK-J4 groups in a 96-well plate for ELISA (Elabscience, Wuhan, China) detection. Levels of the inflammatory mediator, IL-6, were measured by adding samples to wells of a microplate pre-coated with capture antibody to allow IL-6-specific binding. After washing, an IL-6 biotinylated detection antibody was added, followed by HRP-conjugated streptavidin. After incubation and washing, color development was carried out by adding a tetramethylbenzidine (TMB) substrate in the dark and terminated after 15 min with a stop solution. Absorbance at 450 nm was recorded with a microplate spectrophotometer (Bio-Rad, Hercules, CA, USA). The level of IL-6 in each individual sample was then interpolated from a standard calibration curve constructed from serial dilutions of the recombinant cytokine. Each group was analyzed in three biological replicates.

### 2.6. Animals and Ethical Statement

Adult male C57BL/6 mice (weight, 25–30 g, 10–12 weeks old) were commercially acquired from the same batch from Vital River Laboratory Animal Technology Co., Ltd. (Pinghu, China). Animals were maintained in an animal care facility with four mice per cage under specific pathogen-free (SPF) husbandry, with temperature maintained at 21 ± 0.5 °C, relative humidity at 50 ± 5%, and a 12-h light/dark schedule with onset at 7:30 a.m. Mice were given unrestricted access to regular chow and sterile ddH_2_O and were habituated for 5 days prior to the animal trials.

All research procedures received approval from the Ethics Committee of the Laboratory Animal Science Department, Fudan University (Approval Number: 2024-HSYY-419), and were undertaken strictly following the National Institutes of Health Guide for the Care and Use of Laboratory Animals. All necessary steps were adopted to minimize mouse distress and ensure that the quantity of mice euthanized was kept to a minimum.

### 2.7. In Vivo Experimental Grouping and Drug Administration

**Figure 1:**
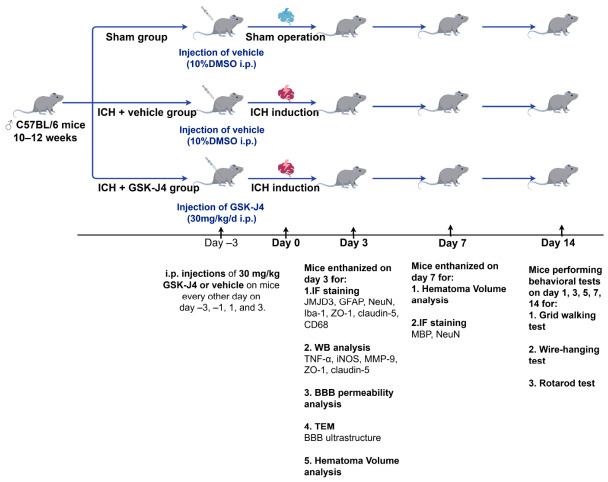
Schematic diagram of the in vivo experimental timeline and animal cohorts. Intra-striatal injection of a sub-lethal dose of collagenase type IV was performed on a stereotaxic frame to establish an intracerebral hemorrhage (ICH) model. Mice in each group received intraperitoneal (i.p.) injections every other day on days −3, −1, 1, and 3 post-surgery. At predetermined post-ICH time intervals, the selected cohorts of mice were euthanized for various analyses. On day 3, cohorts of mice were euthanized via isoflurane overdose, followed by transcardial perfusion. For immunofluorescence (IF) staining and transmission electron microscopy (TEM), brains were collected after pre-fixation, whereas for Western blot (WB) analysis, blood–brain barrier (BBB) permeability analysis and hematoma volume assessment, separate cohorts were used without fixation. On day 7, additional cohorts were euthanized for hematoma volume analysis (without fixation) and IF staining (with pre-fixation). Behavioral tests (grid-walking, wire-hanging, and rotarod tests) were performed on a separate cohort of mice maintained until day 14 post-ICH. This schematic was created with www.figdraw.com, export id: RWYRR44514.

After 5 days of acclimatization, mice were separated using a random number generator into three groups: (1) sham group; (2) ICH + vehicle group; (3) ICH + GSK-J4 group. The ICH model in mice was established by injecting a sub-lethal dose of collagenase type IV into the striatum, whereas sham-operated animals received needle insertion into the striatum without collagenase injection. GSK-J4 was dissolved in 10% DMSO/90% corn oil (*v*/*v*) and administered intraperitoneally (i.p.) at 30 mg/kg/day. Treatment was initiated 3 days before ICH and given on Days −3, −1, 1, and 3. The dosage of GSK-J4 used in this study (30 mg/kg, every other day) was adopted in accordance with previous in vivo studies with modifications to achieve effective concentrations in the brain following ICH [37,45]. Mice in the non-GSK-J4-treated groups were administered 0.5 mL of vehicle (i.p.) on the same schedule. At 3 and 7 days post-ICH, mice were deeply anesthetized with isoflurane until loss of pedal reflexes and then euthanized by cervical dislocation to ensure rapid and humane death. Perihematomal brain tissues were carefully identified and collected at the corresponding time intervals post-ICH for blinded analysis. Briefly, a total of 143 mice were used. Six mice died within 24 h after surgery and were excluded from the analysis. The final numbers for analysis were: Sham group, *n* = 33; ICH group, *n* = 58; ICH + GSK-J4 group, *n* = 46. At 3 days post-ICH, for immunofluorescence staining, 20 sham and 20 ICH mice (*n* = 4 per staining target) were used for JMJD3/GFAP, JMJD3/NeuN, JMJD3/Iba-1, ZO-1/CD31, and claudin-5/CD31 co-labeling, while 8 ICH + GSK-J4 mice were used for ZO-1/CD31 and claudin-5/CD31 co-labeling (*n* = 4 per target); for Western blotting and transmission electron microscopy (TEM), 18 mice (*n* = 3 per group per assay) were used. For BBB permeability analysis, 10 mice from the ICH and ICH + GSK-J4 groups were used at 3 days (*n* = 5 per group). For hematoma volume analysis, 10 mice from the ICH and ICH + GSK-J4 groups were used at 3 days (*n* = 5 per group), and another 10 at 7 days (*n* = 5 per group). At 3 days post-ICH, 10 mice from the ICH and ICH + GSK-J4 groups were selected for CD68/Iba-1 co-immunofluorescence staining (*n* = 5 per group); at 7 days post-ICH, another 10 mice from the same groups were selected for MBP/NeuN co-immunofluorescence staining (*n* = 5 per group). For behavioral tests, 21 mice from the sham, ICH, and ICH + GSK-J4 groups were assessed at 1, 3, 5, 7, and 14 days post-ICH (*n* = 7 per group). The study flowchart is shown in Figure 1.

### 2.8. In Vivo Stereotaxic ICH Model Establishment

Intra-striatal stereotaxic injection of a predetermined sub-lethal dose of collagenase type IV was used to establish an in vivo ICH model. Mice were allocated using a computer-generated randomization schedule into the sham, ICH+ vehicle, or ICH+ GSK-J4 groups. Briefly, anesthesia was induced by placing mice in a box filled with 3% isoflurane for rapid induction. The mice were then secured in a stereotactic apparatus (Stoelting, Wood Dale, IL, USA) with 1.5% isoflurane inhalation through a nose cone for maintenance. Before surgery, the surgical region above the bregma was shaved and sterilized, and the scalp was sectioned along the midline to reveal the skull. The periosteum was gently removed with a sterile cotton swab immersed in hydrogen peroxide, and a 1-mm circular craniotomy was performed at the coordinates overlying the deep ipsilateral striatum, relative to the bregma: 0.5 mm anterior and 2.2 mm lateral. A 10-μL microinjection syringe (model 701, Hamilton Company, Giarmata, Romania) was lowered to a depth of 3.5 mm from the pial surface. A total of 0.0375 U collagenase IV (diluted in 0.5 μL of sterile saline) was infused through the syringe for 5 min. Then, the syringe remained in situ for another 5 min to preclude fluid regurgitation and facilitate diffusion of collagenase IV, and then slowly withdrawn. The small circular bone opening was carefully covered with bone wax (Aesculap AG, Tuttlingen, Germany), and the incision was sutured. After the circular craniotomy, the unconscious mice were laid on a heating blanket (37 °C) until sufficiently recovered. Sham-operated mice received a stereotaxic saline injection into the ipsilateral striatum.

### 2.9. Immunofluorescence Staining

On days 3 and 7 after successfully modeling, the predetermined cohort of mice designated for immunofluorescence analysis was selected. Once deep anesthesia was achieved, the mice confirmed the absence of response to toe pinch and marked respiratory depression, before undergoing transcardial perfusion with ice-cold saline. Then, mice were immediately pre-fixated with 4% paraformaldehyde, followed by thorough post-fixation for 18 h. After fixation, the brains were equilibrated in an ascending sucrose series (15% and 30%) until they sank. For embedding, the brains were placed in small cups filled with OCT compound (Tissue-Tek, Torrance, CA, USA) and preserved at −20 °C. Coronal slices (20 μm) were cut from the frozen tissue, and those passing through the injection site (at the level of Bregma +0.5 mm) were specifically retained for subsequent analysis. The sections of all brain tissues were performed in cryostat (Leica, Wetzlar, Germany).

Before staining, sections were carefully rinsed with PBS via a pipette to eliminate the OCT compound (10 min × 3 times). After excess liquid was carefully blotted away around the sections with tissue paper, we used an immunohistochemistry pen to draw a hydrophobic circle surrounding the brain tissue on the slides. This barrier served to confine the primary antibody solution to the tissue during incubation. Sections were then blocked in a humidified dark chamber with 10% donkey serum at room temperature for 60 min, followed by primary antibody incubation at 4 °C in dark overnight. The donkey serum and primary antibody were prepared immediately before use, diluted in PBS containing 0.3% Triton X-100. For the following immunofluorescence staining, primary antibodies were used at the dilutions indicated below for targeted purposes of immunostaining:

- To evaluate JMJD3 expression in different cerebral cell types at 72 h post-ICH, sections were double-stained for JMJD3 (1:100) and either GFAP (astrocyte marker, 1:600), NeuN (neuron marker, 1:800), or Iba-1 (microglia marker, 1:500);
- To assess BBB disruption at 72 h post-ICH, sections were double-stained for CD31(endothelial marker, 1:100) and either ZO-1 (1:100) or claudin-5 (1:200);
- To evaluate the phagocytic microglia at 3 days post-ICH, sections were double-stained for CD68 (phagocytic activity marker, 1:1000) and Iba-1 (1:500);
- To evaluate the mean white matter intensity and mean neuronal intensity at 7 days post-ICH, sections were double-stained with a myelin sheath marker (MBP, 1:100) and NeuN (1:800).

After incubation, sections were rinsed to remove unbound primary antibodies in PBS (10 min × 3 times). Subsequently, they were incubated in humidified dark chamber for 2 h at room temperature with the following fluorophore-conjugated secondary antibodies (all from Abcam, Cambridge, UK): donkey anti-rabbit Alexa Fluor 488 (Catalogue Number: ab150073; green, 1:500), donkey anti-rabbit Alexa Fluor 555 (Catalogue Number: ab150074; red, 1:500), donkey anti-rat Alexa Fluor 488 (Catalogue Number: ab150153; green, 1:500), donkey anti-rat Alexa Fluor 555 (Catalogue Number: ab150154; red, 1:500), and donkey anti-goat Alexa Fluor 555 (Catalogue Number: ab150130; red, 1:500). Sections were then coverslipped with DAPI mounting medium containing an anti-fade agent (Catalogue Number: ab104139; blue, used as supplied) for nuclear labeling. Micrographs were imaged using a Nikon C2plus confocal laser scanning microscope (Tokyo, Japan).

### 2.10. Quantitative Image Analyses of Fluorescence

Quantitative analyses of fluorescence were performed within the regions of interest (ROIs). Each ROI was captured using a 40× objective lens (numerical aperture, NA 1.0) designed for aqueous media at a resolution of 1024 × 1024 pixels, corresponding to a field area of 318.5 μm × 318.5 μm (pixel size: 0.311 μm). ROIs were acquired using the same laser and detector settings and were analyzed as follows:

#### 2.10.1. Method for Quantification of JMJD3 Expression in Different Brain Cell Types

To evaluate JMJD3 expression in astrocytes, neurons and microglia at 72 h post-ICH, for each mouse, one coronal brain section corresponding to Bregma +0.5 mm (the region of the maximal hematomal area of the striatum) was selected for analysis. Three ROIs were systematically selected in the perihematomal tissue around the hematoma boundary per brain section. These ROIs were localized using vertical and horizontal lines through the center of the hematoma, representing the lateral, superior, and inferior aspects of the perihematomal region, respectively. Anatomically matched regions were analyzed in the sham group.

For each ROI, the following cell types were manually counted by an investigator blinded to the group allocations: GFAP^+^ astrocytes, NeuN^+^ neurons, Iba-1^+^ microglia, JMJD3^+^/GFAP^+^ astrocytes, JMJD3^+^/NeuN^+^ neurons, and JMJD3^+^/Iba-1^+^ microglia. And using Image J software (version 1.53t; NIH, Bethesda, MD, USA), the total fluorescence density of JMJD3 within all JMJD3^+^ astrocytes and the total area of JMJD3^+^/GFAP^+^ astrocytes were both quantified. The same analysis was performed for neurons. These values were then used to calculate the cell type-specific mean fluorescence intensity (MFI).

On the basis of these counts, the following three parameters were calculated for astrocytes and neurons:

- Percentage of JMJD3^+^ astrocytes (or neurons) relative to the total number of astrocytes (or neurons) (%) to determine the proportion of a given cell type expressing JMJD3;
- Number of JMJD3^+^ astrocytes (or neurons) per unit area (per mm^2^) to quantify the spatial density of JMJD3-expressing-cells;
- MFI of JMJD3 in astrocytes (or neurons), measured with ImageJ (version 1.53t) and defined as the total fluorescence density within all JMJD3^+^ cells (astrocytes or neurons) divided by the total area of the corresponding JMJD3^+^ cell population (a.u.), was used to assess the single-cell expression level.

For microglia, the number of JMJD3^+^Iba-1^+^ double-positive cells per mm^2^ was quantified.

For each parameter, the average of three ROIs per section was calculated for statistical analysis. Four mice per group were used for analyses.

#### 2.10.2. Method for Quantification of Tight Junction Protein Expression in BBB

To investigate alterations in the BBB at the molecular level, the fluorescence expression of zonula occludens-1 (ZO-1) and claudin-5, two major structural tight junction (TJ) proteins in cerebral vasculature, was quantified in the ipsilateral striatum among the sham, ICH, and ICH + GSK-J4-treated mice at the acute phase (72 h) after ICH. For each mouse, one coronal brain section at the level of the maximal hematoma area (Bregma +0.5 mm) was selected. One vascular ring-containing region was selected from the lateral, superior, and inferior aspects of the perihematomal area, and designated as three ROIs for imaging and analysis. In the sham group, anatomically comparable vascular areas were analyzed. The abundance of ZO-1 was quantified by calculating the area of ZO-1^+^ signal colocalized with CD31, normalized to the total CD31^+^ vascular area (×100%). The same procedure was performed on adjacent sections for claudin-5 quantification. For each animal, the average of three ROIs per section was calculated for statistical analysis. Four mice per group were used for the analyses.

#### 2.10.3. Method for Quantification of Phagocytic Microglia

To evaluate alterations in phagocytic microglia in the peri-hematomal area at 3 days post-ICH with or without GSK-J4 treatment, the numbers of CD68^+^ (phagocytic activity marker) and Iba-1^+^ (microglia marker) double-positive cells were assessed. For each mouse, one coronal brain section at the level of the maximal hematoma area (Bregma +0.5 mm) was selected and three pre-defined ROIs were selected from the lateral, superior, and inferior aspects of the perihematomal area. In ICH and ICH + GSK-J4 groups, CD68^+^/Iba-1^+^ microglia per unit area (cells/mm^2^) were counted in each ROI, and the numbers from three ROIs per section were averaged per section. Five mice per group were used for the analyses.

#### 2.10.4. Method for Quantification of White Matter and Neuronal Recovery

To evaluate alterations of white matter and neuronal tissue at 7 days post-ICH with or without GSK-J4 treatment. The expressions of the white matter marker MBP and neuronal marker NeuN were separately assessed. For each mouse, coronal brain sections at the level of the Bregma +0.5 mm were selected. Two ROIs were selected as follows: (1) ROIs in striatum (STR), selected from the perihematomal striatum lateral to the hematoma boundary and (2) ROIs in the ipsilateral cortex (CTX), selected at the same coronal level of the ROIs in striatum. In each ROI, the MFI of MBP and NeuN was measured separately using ImageJ software (version 1.53t). For each mouse and staining marker, the MFI values for each ROI in two serial sections were averaged to minimize sampling bias and account for regional heterogeneity. Five mice per group were used for the analyses.

### 2.11. Hematoma Volume Analysis

To more intuitively assess hematoma resolution, two cohorts of mice were euthanized by isoflurane overdose at 3 days and 7 days post-ICH. Mice were confirmed by abolished reflex and markedly slowed respiration before being transcardially perfused with ice-cold saline. Following established methods [46], after cervical dislocation, mouse brains were rapidly removed, chilled at −20 °C for 20 min, and placed in a brain matrix (RWD Life Science, Shenzhen, China) for coronal sectioning according to the designated orientation. Coronal brain slices were cut at a thickness according to the slot size of the matrix (1 mm in this study). The brain slices were sequentially arranged and imaged. We delineated the hematoma boundary in each section, and the hematoma volume was calculated as follows [47]:

Hematoma volume (mm^3^) = Σ (hematoma area per section [mm^2^]) × section thickness (1 mm)

### 2.12. Western Blot Analysis

On three days post-ICH, a cohort of mice were euthanized and the hemispheres containing the hematoma were harvested. The striata were carefully dissected for total protein extraction. Radioimmunoprecipitation assay lysis buffer (Catalogue Number: MB-030-0050; Rockland Immunochemicals, Pottstown, PA, USA) was used as a protein homogenizer and the homogenized proteins were assayed using a BCA Protein Quantification Kit (Catalogue Number: 20201ES; Yeasen, Shanghai, China). After mixing with SDS loading buffer, the protein lysates were boiled in a ThermoCell metal bath (Bioer, Hangzhou, China) for 5 min to achieve denaturation. Aliquots containing 30 μg of protein per lane were loaded into the wells of 10% or 12% SDS-PAGE gels, with a protein marker loaded in a separate lane. The proteins were electrophoresed until all marker bands were fully separated, and then were quickly electrotransferred by eBlot L1 Quick wet converter (GenScript, Nanjing, China) onto a methanol-activated PVDF membranes (Starvio, Shanghai, China). After blocking the nonspecific protein binding sites with 5% skim milk in TBST (Tris-buffered saline containing 0.1% Tween 20) for 2 h at room temperature, membranes are probed overnight at 4 °C with primary antibodies, including: anti-JMJD3 (1:1000), anti-iNOS (1:500), anti-TNF-α (1:1000), anti-MMP-9 (1:500), anti-ZO-1 (1:1000), anti-claudin-5 (1:1000), and anti-β-actin (1:10,000). On the second day, after washing with TBST (5 min × 4 times), membranes were then probed with HRP-conjugated secondary antibodies (1:5000; Proteintech, Wuhan, China) for 2 h at room temperature. The bands of interest were visualized by enhanced chemiluminescence (Thermo Fisher, Waltham, MA, USA). Densitometric quantification of target protein bands was executed using ImageJ (version 1.53t), with β-actin serving as the reference protein control. Relative protein expression levels in the ICH-vehicle and ICH + GSK-J4 groups were normalized to the sham group. Three mice per group were used for analyses.

### 2.13. Evaluation of BBB Permeability

Evans blue (EB) staining was performed on day 3 post-ICH to functionally evaluate BBB permeability. Briefly, 2% EB dye (Catalog No. ST3273, Beyotime Biotechnology, Haimen, China) dissolved in normal saline was intravenously administered into the femoral vein at a dose of 4 mL/kg. After 2 h of circulation, the mice were deeply anesthetized, and a transcardial perfusion was performed slowly with ice-cold saline until the fluid exiting the right atrium became clear. The whole brains were then rapidly removed, placed in a brain matrix, and cut into 1-mm-thick coronal slices. The slices were sequentially arranged and photographed. For quantitative analysis, the brain slices from each mouse were separately collected, weighed, and homogenized in 4 mL of PBS. The homogenates were mixed with an equal volume of 50% trichloroacetic acid and incubated overnight at 4 °C. After centrifugation at 12,000× *g* for 20 min, the supernatant was collected, and its absorbance at 620 nm was measured using a microplate reader (Biotek, Winooski, VT, USA). The Evans blue concentration (μg/g brain tissue) was calculated using the following formula [48]:

EB concentration (μg/g) = EB concentration in supernatant (μg/mL) × total volume of extraction solution (mL)/brain tissue wet weight (g)

### 2.14. Transmission Electron Microscopy (TEM)

At 3 days after ICH surgery, transmission electron microscopy (TEM) was utilized for assessing the brain microvascular ultrastructure after ICH induction, focusing on endothelial cell morphology, tight junction integrity, and basement membrane thickness. Briefly, brain tissue samples (1 mm^3^) collected from the perihematomal region were fixed in 4% glutaraldehyde formulated in phosphate buffer (0.1 M) at 4 °C for 16 h. Following fixation, the specimens were immersed in 1% osmium tetroxide for 2 h and subsequently dehydrated through an ascending ethanol series, infiltrated with propylene oxide, and encapsulated for subsequent sectioning by using Embed 812 resin (Electron Microscopy Sciences, Morgantown, PA, USA). Ultrathin Sections (70 nm) were sliced with an ultramicrotome (Leica EM UC7, Wetzlar, Germany) and picked up onto copper grids. The sections were then incubated sequentially by inverting the grids on drops of uranyl acetate and lead citrate. Finally, after rinsing with ddH_2_O and blotting dry, the sections were examined under a Talos L120C transmission electron microscope (Thermo Fisher Scientific, Waltham, MA, USA).

### 2.15. Behavioral Assessments

Sensorimotor functions were evaluated at baseline (pre-ICH) and on day 1, 3, 5, 7, and 14 post-ICH using three validated tests [49,50]:

#### 2.15.1. Grid Walking Test

Mice were positioned on a rectangular metal wire grid (2 cm × 2 cm) and permitted to freely walk for 2 min. A “foot fault” was counted if a forepaw or a hindpaw slipped between the grid bars. The foot fault rates are expressed as follows: (numbers of foot faults/total number of steps) × 100%. Five to seven mice per group contributed to the data, which are expressed as the mean ± standard error of the mean (SEM). All sensorimotor functional tests and data processing were executed in a blinded way.

#### 2.15.2. Wire-Hanging Test

Mice were hung by their forelimbs on a 55 cm-long iron wire that was suspended between two platforms (50 cm high), and their performance was scored on a 0–5 scale, as follows: 0 = immediate fall; 1 = holds on with one forelimb; 2 = holds on with both forelimbs; 3 = attempts to climb with one hindlimb; 4 = attempts to climb with both hindlimbs; and 5 = successfully climbs to either platform. Each mouse underwent three trials with two 10-min intervals, and the median score was used for analysis. Five to seven mice per group contributed to the data, which were expressed as median with interquartile range (IQR). All sensorimotor functional tests and data processing were executed in a blinded way.

#### 2.15.3. Rotarod Test

Mice were manually set on a rotarod system (Ugo Basile, Varese, Italy) starting at 5 rpm, with acceleration of 1 rpm every 10 s. The latency to fall was measured as the time the mouse stayed on the accelerating rotarod. Each mouse underwent three attempts with two 10-min intervals, and the latency per mouse was averaged for analysis. Five to seven mice per group contributed to the data, which were expressed as the mean ± SEM. All sensorimotor functional tests and data processing were executed in a blinded way.

### 2.16. Statistical Analysis

All statistical analyses were performed using GraphPad Prism 8.0 software (GraphPad Software, San Diego, CA, USA). The Shapiro–Wilk test was used to check for normal distribution. For normally distributed data, comparisons between two independent groups were analyzed using two-tailed unpaired Student’s *t*-test, and comparisons between three or more groups were analyzed using one-way or two-way analysis of variance (ANOVA) followed by Sidak’s post hoc test, and data are presented as mean ± SEM. For non-normally distributed data, differences between two independent groups were analyzed by the Wilcoxon Rank-Sum Test, and data are presented as median (interquartile range, IQR). *p*-value < 0.05 was considered statistically significant.

## 3. Results

### 3.1. Jmjd3 Is Upregulated in Hemin-Induced Astrocyte and Correlates with Neuroinflammatory Signaling In Vitro

**Figure 2:**
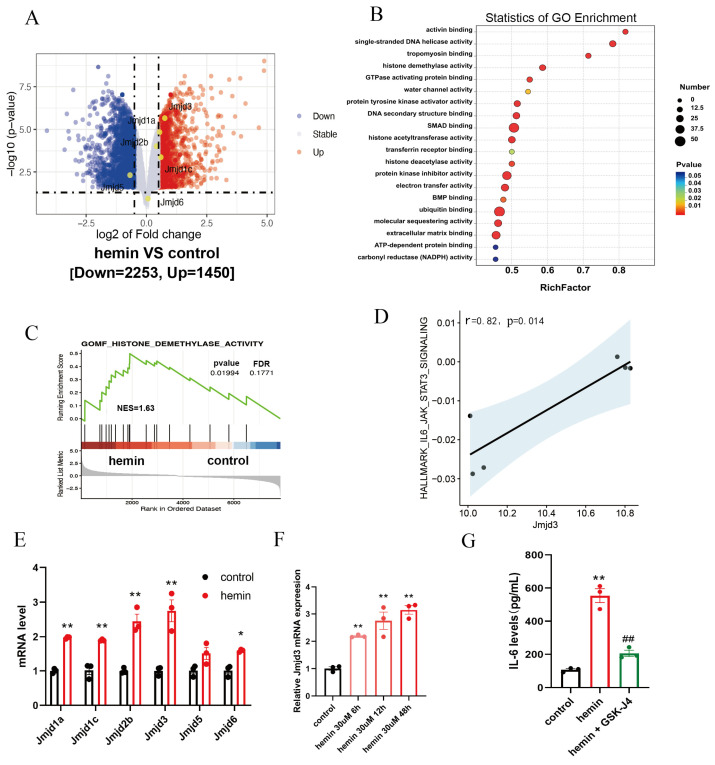
*Jmjd3* is upregulated in hemin-induced astrocyte and correlates with neuroinflammation in vitro. *Jmjd3* (Jumonji C domain-containing protein 3, also known as KDM6B) is a histone H3 lysine 27 demethylase. (**A**–**D**) The regulation of histone demethylase genes is analyzed by RNA-sequencing in hemin-treated C8-D1A astrocyte cell line at 12 h post-induction, compared with controls. (**A**) Differentially expressed genes (DEGs) are visualized in the volcano plot between groups (*n* = 3 per group). Using thresholds of fold change >1.5 or <0.67 and adjusted *p* < 0.05 as DEGs, upregulated and downregulated genes are identified and displayed in red and blue, respectively. Gray dots indicate genes with no significant change. Histone demethylase family genes (*Jmjd1a*, *Jmjd1c*, *Jmjd2b*, *Jmjd3*, *Jmjd5*, and *Jmjd6*) are highlighted in yellow dots, with *Jmjd3* identified as a top-upregulated gene. (**B**) To investigate the functional characteristics of upregulated genes in hemin-stimulated C8-D1A astrocytes, gene ontology GO enrichment analysis is performed focusing on molecular function terms. The top 20 significantly enriched items are shown, with “histone demethylase activity” highlighted (red line). (**C**) Enrichment of the histone demethylase activity GO term of molecular function in hemin-treated vs. vehicle-treated astrocytes, revealed by Gene Set Enrichment Analysis (GSEA). NES and FDR values are shown. (**D**) Scatter plot with a fitted curve reflects the correlation between *Jmjd3* gene expression and the interleukin-6 (IL-6)/JAK/STAT3 pro-inflammatory hallmark pathway in control and hemin-treated astrocytes analyzed by gene set variation analysis (GSVA) (n = 3 per group). Pearson correlation analysis demonstrated a significant positive correlation coefficient (r = 0.82, *p* = 0.014). (**E**,**F**) Quantitative real-time polymerase chain reaction (RT-qPCR) confirms the differential expression of histone demethylase genes identified by RNA-seq. (**E**) mRNA levels of histone demethylase family genes in hemin-treated vs. vehicle-treated astrocytes at 12 h post-treatment. (**F**) Time-course analysis of *Jmjd3* mRNA transcription in hemin-treated astrocytes at 6, 12, and 48 h post-treatment vs. vehicle-treated astrocytes. Transcription levels are normalized to glyceraldehyde-3-phosphate dehydrogenase *(Gapdh)* and calibrated to the vehicle-treated group, and are presented as fold changes. Statistically, two-way ANOVA (**E**) or one-way ANOVA (**F**) are used for the initial analysis, and *p*-values from predefined pairwise comparisons (hemin vs. controls) are obtained following Sidak’s post hoc test. All values are presented as mean ± standard error of the mean (SEM) from *n* = 3 biological replicates. * *p* < 0.05, ** *p* < 0.01 vs. control group. (**G**) Enzyme-linked immunosorbent assay (ELISA) evaluates the anti-inflammatory effects of JMJD3 inhibition by GSK-J4 onIL-6 secretion (pg/mL) in hemin-induced astrocytes after 12 h of induction. Astrocytes were pretreated with GSK-J4 (5 μM) for 30 min, then stimulated with hemin (30 μM) for 12 h. Statistically, one-way ANOVA is used for the initial analysis, and *p*-values from predefined pairwise comparisons (sham vs. ICH, ICH vs. ICH + GSK-J4) are obtained following Sidak’s post hoc test. All values are presented as mean ± SEM from *n* = 3 biological replicates. ** *p* < 0.01 vs. control group; ## *p* < 0.01 vs. hemin group.

To identify histone demethylase genes implicated in astrocyte activation under ICH pathological conditions, we performed RNA sequencing on C8-D1A astrocytes treated with 30 μM hemin for 12 h (an in vitro ICH model mimicking heme toxicity). Differentially expressed gene (DEG) analysis revealed 3703 genes with altered transcription in hemin-treated astrocytes relative to vehicle-treated controls, comprising 1450 up-modulated and 2253 down-modulated genes, as depicted in the volcano plot (Figure 2A). Gene Ontology (GO) enrichment analysis of molecular functions identified histone demethylase activity as one of the most significantly enriched terms (Figure 2B), indicating a widespread dysregulation of histone demethylation in astrocytes exposed to ICH-related stress. Gene Set Enrichment Analysis (GSEA) also validated that histone demethylase activity-related gene sets are significantly enriched in hemin-treated astrocytes (normalized enrichment score [NES] = 1.63, FDR = 0.177; Figure 2C). Among the DEGs in the volcano plot, *Jmjd3* was highlighted as a top-regulated histone demethylase gene (fold change = 1.7, adjusted *p* < 0.001; Figure 2A), and there was a strong positive association between *Jmjd3* expression and the IL-6/JAK/STAT3 pro-inflammatory signaling pathway, as determined by Pearson correlation analysis (r = 0.82, *p* = 0.014; Figure 2D), suggesting a functional link between *Jmjd3* and astrocytic pro-inflammatory signals in vitro.

RT-qPCR confirmed the RNA-seq findings and further characterized the relative transcriptive levels of *Jmjd3* and other histone demethylases: *Jmjd3* mRNA levels were increased to 2.8 ± 0.3-fold in hemin-treated astrocytes, a magnitude of upregulation far exceeding that of *Jmjd1a* (2.0 ± 0.1-fold), *Jmjd2b* (2.5 ± 0.2-fold), and other histone demethylase gene family members (*p* < 0.001 for these three genes; *n* = 3 per group, two-way ANOVA for the initial analysis, and Sidak’s post hoc test for predefined pairwise comparisons, hemin vs. control; Figure 2E). A time-course analysis revealed that *Jmjd3* mRNA transcription was dynamically upregulated after hemin stimulation, with significant increases to 2.2 ± 0.1-fold at 6 h, 2.8 ± 0.3-fold at 12 h, and a 3.2 ± 0.2-fold at 48 h post-treatment (*p* = 0.005 for 6 h, *p* < 0.001 for 12 h and 48 h; *n* = 3 per group, one-way ANOVA for the initial analysis, and Sidak’s post hoc test for predefined pairwise comparisons, hemin vs. control), indicating sustained elevation throughout the 48 h period (Figure 2F). To verify the causal role of JMJD3 in astrocytic neuroinflammation, we pre-treated C8-D1A cells with 5 μM GSK-J4 (a specific JMJD3 inhibitor) 30 min prior to hemin exposure. ELISA showed that after 12 h of hemin (30 μM) exposure, IL-6 secretion in astrocytes was elevated to 5.2 ± 0.5-fold (*p* < 0.001 vs. control group; *n* = 3 per group, one-way ANOVA for the initial analysis, and Sidak’s post hoc test for predefined pairwise comparisons, hemin vs. control), and GSK-J4 pre-treatment reduced hemin-induced IL-6 secretion by 63 ± 4% (*p* < 0.001 vs. hemin group; *n* = 3 per group, one-way ANOVA for the initial analysis, and Sidak’s post hoc test for predefined pairwise comparisons, hemin vs. hemin + GSK-J4; Figure 2G). Collectively, these in vitro data demonstrate that *Jmjd3* is a key dysregulated histone demethylase gene in hemin-induced astrocytes and operates as a critical modulator of astrocytic pro-inflammatory signaling in the context of ICH.

### 3.2. JMJD3 Is Upregulated in Astrocytes in the Perihematomal Region Following Acute ICH In Vivo

**Figure 3:**
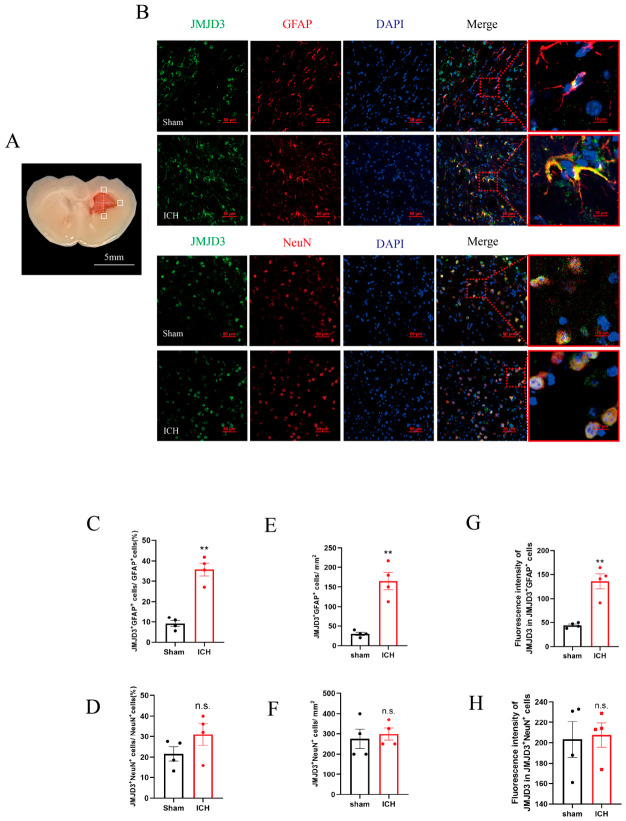
JMJD3 is upregulated in astrocytes in the perihematomal region during the acute phase of the ICH mouse model. (**A**) Cell-specific regulation of JMJD3 protein was assessed by double immunofluorescence staining and quantitative image analysis in collagenase-induced ICH mice at three days post-ICH compared with sham-operated controls. The diagram illustrates a representative coronal brain section taken through the injection site (Bregma +0.5 mm) to show the maximal hematoma cross-sectional area. Three regions of interest (ROIs) were systematically selected for fluorescence intensity measurement: one lateral to the hematoma along the horizontal line, one superior to the hematoma along the vertical line, and one inferior to the hematoma along the vertical line. These ROIs are indicated by boxed areas. The horizontal and vertical lines are indicated by dashed lines crossing the hematoma center. Scale bars: 5 mm. (**B**) Typical immunostaining images of JMJD3 (green) colocalized with glial fibrillary acidic protein (GFAP, astrocyte marker, red) and neuron-specific nuclear protein (NeuN, neuronal marker, red) in sham and ICH mice at three days post-ICH. Enlarged views of the boxed regions reveal representative astrocytes and neurons. In the ICH group, GFAP^+^ astrocytes with high JMJD3 expression were detected, whereas the sham group showed only GFAP^+^ astrocytes with low JMJD3 expression. JMJD3^+^/NeuN^+^ double-positive neurons were observed in both groups. Scale bars: 50 μm (insets: 10 μm). (**C**,**D**) Quantitative analysis of the percentage of JMJD3^+^ astrocytes among total astrocytes (**C**) and the percentage of JMJD3^+^ neurons among total neurons (**D**) in the perihematomal region of ICH and sham mice. (**E**,**F**) Quantitative analysis of the number of JMJD3^+^ astrocytes per mm^2^ (**E**) and the number of JMJD3^+^ neurons per mm^2^ (**F**) in the perihematomal region of ICH and sham mice. (**G**,**H**) Quantitative analysis of mean JMJD3 fluorescence intensity in JMJD3^+^/GFAP^+^ cells (a.u.) (**G**) and JMJD3^+^/NeuN^+^ cells (a.u.) (**H**) In the perihematomal region of ICH and sham mice. For each parameter, values were averaged from three ROIs per mouse. Two-tailed unpaired Student’s *t*-test is used for analysis (sham vs. ICH). All values are presented as mean ± SEM from *n* = 4 animals per group. ** *p* < 0.01, n.s. not significant.

To validate the in vitro observations and determine the cellular localization of JMJD3 after ICH, immunofluorescence staining and quantitative analysis were performed on frozen brain sections from collagenase-induced ICH mice at three days post-injury (a critical time point for acute secondary injury). We drew horizontal and vertical lines through the hematoma center and selected three regions of interest (ROIs) at their intersections within the perihematomal region: one ROI lateral to the hematoma representing the lateral perihematomal region, one ROI superior to the hematoma representing the superior perihematomal region, and one ROI inferior to the hematoma representing the inferior perihematomal region (Figure 3A). We analyzed JMJD3 expression in GFAP^+^ astrocytes and NeuN^+^ neurons using three complementary parameters in each ROI: (1) the percentage of JMJD3^+^ astrocytes (or neurons) among the total astrocytes (or neurons), reflecting the proportion of JMJD3^+^ cells in each population after ICH; (2) the number of JMJD3^+^ astrocytes (or neurons) per unit area (/mm^2^), reflecting the spatial density of JMJD3^+^ astrocytes or neurons; and (3) the mean fluorescence intensity (MFI) of JMJD3 in double-positive astrocytes (or double-positive neurons), reflecting JMJD3 expression at the single-cell level. For each parameter, the values from the three systematically selected ROIs were averaged for statistical analysis to minimize regional astrocyte heterogeneity.

The results showed that in sham-operated mice, JMJD3 protein was predominantly expressed in neurons (NeuN^+^ cells) with low expression detected in astrocytes (GFAP^+^ cells) in the striatal region (Figure 3B). In contrast, ICH mice exhibited marked upregulation of JMJD3 in the perihematomal striatum, with a striking increase in JMJD3 colocalization with the astrocyte marker GFAP (Figure 3B). Quantitative morphometric analysis confirmed these phenotypic changes; for example, in the ICH group, the proportion of JMJD3^+^/GFAP^+^ astrocytes increased to 3.9 ± 0.7-fold (*p* < 0.001, two-tailed unpaired Student’s *t*-test, *n* = 4 per group; Figure 3C) compared to that in the sham group, and the spatial density of double-positive astrocytes also increased to 5.5 ± 1.1-fold (*p* = 0.001, two-tailed unpaired Student’s *t*-test, *n* = 4 per group; Figure 3E), and similarly, MFI of JMJD3 in JMJD3^+^/GFAP^+^ astrocytes increased to 3.1 ± 0.4-fold (*p* = 0.001, two-tailed unpaired Student’s *t*-test, *n* = 4 per group; Figure 3G).

Notably, JMJD3 expression in neurons was not altered by in vivo ICH induction in the percentage of JMJD3^+^/NeuN^+^ double-positive neurons (1.4 ± 0.2-fold, *p* = 0.184, two-tailed unpaired Student’s *t*-test, *n* = 4 per group; Figure 3D), in the spatial density of JMJD3^+^/NeuN^+^ cells (1.1 ± 0.1-fold, *p* = 0.688, two-tailed unpaired Student’s *t*-test, *n* = 4 per group; Figure 3F), or in the MFI of JMJD3 in JMJD3^+^ neurons (*p* = 0.865, two-tailed unpaired Student’s *t*-test, 1.0 ± 0.1-fold, *n* = 4 per group; Figure 3H). We also examined expression in other glial cells like microglia and found an increase in JMJD3 expression in Iba-1^+^ microglia in the perihematomal region at day 3 post-ICH (Supplementary Figure S1). These in vivo results demonstrate upregulation of JMJD3 in astrocytes and microglia but not in neurons in the acute stage of ICH, confirming astrocytes as a key cellular source of JMJD3-mediated pathological signaling after hemorrhagic stroke.

### 3.3. JMJD3 Inhibition Attenuates Neuroinflammation and Subsequently Preserves Blood–Brain Barrier Integrity in ICH Mice

**Figure 4:**
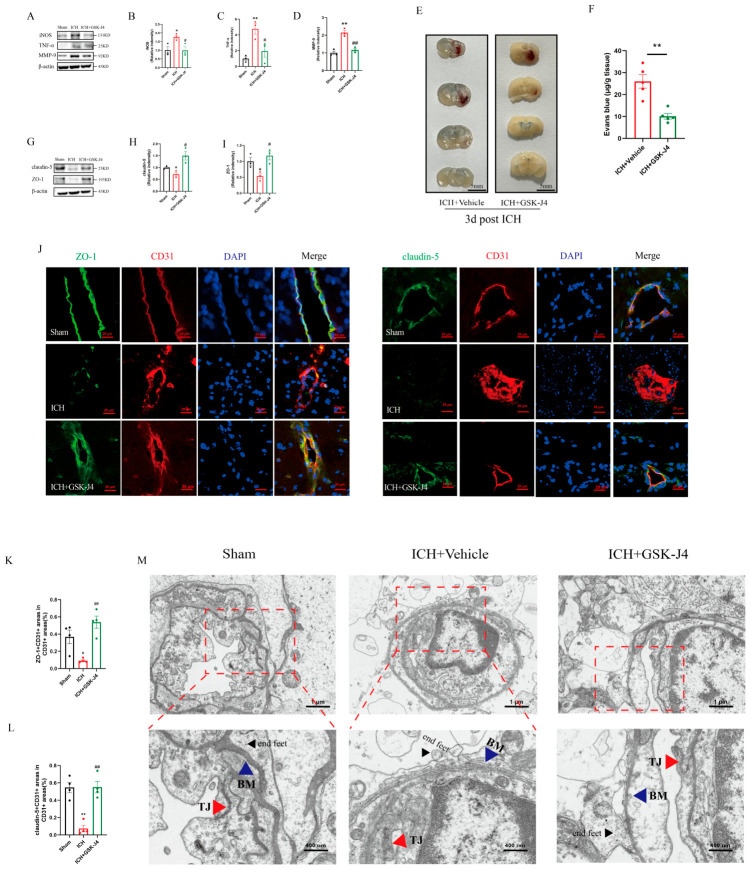
JMJD3 inhibition attenuates neuroinflammation, reduces BBB permeability, and preserves BBB integrity in acute ICH mouse model. (**A**) Effects of GSK-J4 on the alterations of pro-inflammatory mediator in the collagenase-induced ICH mice were evaluated at 3 days post-induction. Protein levels of inducible nitric oxide synthase (iNOS), tumor necrosis factor-alpha (TNF-α), and matrix metalloproteinase-9 (MMP-9) in the striatum ipsilateral to the hematoma was measured by Western blot analysis in sham, ICH, and ICH + GSK-J4 group. Mice were administered i.p. injections of 30 mg/kg GSK-J4 on days −3, −1, 1, and 3; ICH was induced on day 0. Representative blots are shown. (**B**–**D**) Densitometric quantification of the bands for (**B**) iNOS, (**C**) TNF-α, and (**D**) MMP-9 was executed using ImageJ. Protein levels are normalized to loading control β-actin, and are calibrated to sham groups and presented as fold changes. Two-tailed unpaired Student’s *t*-test is used for analysis (sham vs. ICH, ICH vs. ICH + GSK-J4). All values are presented as mean ± SEM from *n* = 3 animals per group. * *p* < 0.05, ** *p* < 0.01 vs. sham group; # *p* < 0.05, ## *p* < 0.01 vs. ICH group. (**E**,**F**) Effects of GSK-J4 on reducing BBB permeability in collagenase-induced ICH mice at 3 days post-induction were examined by Evans blue (EB) extravasation assays. Representative pictures show the extravasation of EB dyes in the ICH and ICH + GSK-J4 groups (**E**), and then the quantitative analysis of EB leakage was performed (**F**). Scale bars: 5 mm. The content of EB (μg/g brain tissue) was calculated using the formula: EB concentration (μg/mL) × total volume of extraction solution (mL)/brain tissue wet weight (g). Two-tailed unpaired Student’s *t*-test is used for analysis (ICH vs. ICH + GSK-J4). All values are presented as mean ± SEM from *n* = 5 animals per group. ** *p* < 0.01 vs. ICH group. (**G**) Effects of GSK-J4 on the alterations of tight junction proteins in the collagenase-induced ICH mice were evaluated at 3 days post-induction. Protein expression of claudin-5 and zonula occludens protein-1(ZO-1) in the striatum ipsilateral to the hematoma was measured by Western blot analysis in sham, ICH, and ICH + GSK-J4 group. Representative blots are shown. (**H**,**I**) Densitometric quantification of the bands for (**H**) claudin-5 and (**I**) ZO-1 was executed using ImageJ. Protein levels were normalized to β-actin, and are calibrated to sham groups and presented as fold changes. Two-tailed unpaired Student’s *t*-test is used for analysis (sham vs. ICH, ICH vs. ICH + GSK-J4). All values are presented as mean ± SEM from *n* = 3 animals per group. * *p* < 0.05 vs. sham group; # *p* < 0.05 vs. ICH group. (**J**) To further evaluate the morphological changes in tight junction protein after GSK-J4 administration following ICH induction, we applied immunofluorescence staining on frozen brain slices from sham, ICH, and ICH + GSK-J4 mice at 3 days post-ICH. Representative images show the colocalization of ZO-1 (green) or claudin-5 (green) with the endothelial marker platelet endothelial cell adhesion molecule 1 (CD31, red) in the striatum ipsilateral to the hematoma. In sham mice, ZO-1^+^/CD31^+^ and claudin-5^+^/CD31^+^ vessels exhibit continuous, circularized staining, and in contrast, ICH mice show discontinuous, fragmented expression of both ZO-1^+^ and claudin-5^+^ along CD31^+^ vessels. GSK-J4 treatment reversed these abnormalities, restoring the linear and continuous distribution of ZO-1^+^ and claudin-5^+^ signals. Scale bars: 20 μm. (**K**,**L**) Quantitative analysis of the ZO-1^+^/CD31^+^ (**K**) and claudin-5^+^/CD31^+^ (**L**) areas a percentage of total CD31^+^ area in the striatum ipsilateral to the hematoma in sham, ICH, and ICH + GSK-J4 mice. Statistically, one-way ANOVA was used for the initial analysis, and *p*-values from predefined pairwise comparisons (sham vs. ICH, ICH vs. ICH + GSK-J4) were obtained following Sidak’s post hoc test. All values are presented as mean ± SEM from *n* = 4 animals per group. * *p* < 0.05, ** *p* < 0.01 vs. sham group; ## *p* < 0.01 vs. ICH group. (**M**) Effects of GSK-J4 on BBB ultrastructure in the perihematomal region at 3 days post-ICH. TEM images showing representative ultrastructure of tight junctions (TJs), basement membrane (BM), and astrocyte endfeet. Upper panels: overview of the sham, ICH, and ICH + GSK-J4 groups. Lower panels: higher-magnification views of the boxed areas to reveal detailed morphology. In the sham group, well-formed TJs (red arrows), an intact and continuous BM (blue arrows), and normal astrocyte endfeet (black arrows) are observed. In the ICH group, these structures are severely disrupted: TJs are highly indistinct (red arrows), the BM is thinned and discontinuous (blue arrows), and astrocyte endfeet are markedly swollen (black arrows). GSK-J4 treatment attenuates these morphological changes, as evidenced by partially restored TJs (red arrows), a more continuous BM (blue arrows), and reduced endfeet swelling (black arrows), indicating a trend toward structural recovery. Red arrows: tight junctions; blue arrows: basement membrane; black arrows: astrocyte endfeet. Scale bars: upper panels, 1 μm; lower panels, 400 nm.

To explore the therapeutic potential of JMJD3 inhibition through evaluation of secondary injury-related phenotypes after ICH, we administered GSK-J4 (30 mg/kg, i.p.) to ICH mice and analyzed the alterations of pro-inflammatory mediators and BBB integrity at three days after ICH. Western blot analysis of perihematomal brain tissues showed that ICH induced robust upregulation of pro-inflammatory mediators. For instance, iNOS protein levels were increased to 1.8 ± 0.1-fold (*p* = 0.027 vs. sham, two-tailed unpaired Student’s *t*-test, *n* = 3 per group), as well as TNF-α levels to 4.8 ± 0.7-fold (*p* = 0.006 vs. sham, two-tailed unpaired Student’s *t*-test, *n* = 3 per group), respectively, in vehicle-treated ICH mice (Figure 4A–C). MMP-9, a pro-inflammatory enzyme that directly degrades BBB structural components, was also upregulated to 2.1 ± 0.1-fold in ICH mice (*p* = 0.003 vs. sham, two-tailed unpaired Student’s *t*-test, *n* = 3 per group; Figure 4A,D). GSK-J4 treatment significantly suppressed this ICH-induced pro-inflammatory response: iNOS and TNF-α levels were reduced by 44 ± 12% (*p* = 0.028, two-tailed unpaired Student’s *t*-test, *n* = 3 per group) and by 60 ± 17% (*p* = 0.049, two-tailed unpaired Student’s *t*-test, *n* = 3 per group), respectively, and MMP-9 expression was decreased by 46 ± 5% (*p* = 0.004, two-tailed unpaired Student’s *t*-test, *n* = 3 per group) compared to vehicle-treated ICH mice (Figure 4A–D). The full, uncropped blots are provided in Supplementary Figure S2. These data confirmed that pharmacological inhibition of JMJD3 effectively attenuated acute neuroinflammation in the perihematomal region after ICH.

Given the central status of BBB disruption in ICH-induced secondary injury, we next examined the effect of GSK-J4 on BBB permeability 3 days post-ICH using an Evans blue (EB) extravasation assay. Representative brain section showed obvious EB extravasation through the disrupted BBB in ICH mice, with a large area of EB dye exudation into the brain parenchyma. In contrast, GSK-J4 administration significantly reduced the area of EB extravasation (Figure 4E), indicating that GSK-J4 reduces BBB permeability. Quantitative analysis confirmed that EB leakage in the ICH + GSK-J4 group was decreased by 61 ± 7% compared to the ICH group (*p* = 0.001, two-tailed unpaired Student’s *t*-test, *n* = 5 per group; Figure 4F). Together, these results demonstrate that GSK-J4 improves BBB function in the acute phase of ICH.

To further assess the structural integrity of BBB, we performed Western blot analysis, immunofluorescence staining and TEM to evaluate tight junction (TJ) protein expression and its peri-vascular distribution, as well as BBB ultrastructure. Western blot analysis showed that vehicle-treated ICH mice exhibited a decline of core TJ proteins: ZO-1 levels were reduced by 46 ± 11% (*p* = 0.023 vs. sham, two-tailed unpaired Student’s *t*-test, *n* = 3 per group) and claudin-5 levels by 28 ± 9% (*p* = 0.037 vs. sham, two-tailed unpaired Student’s *t*-test, *n* = 3 per group) at three days after ICH (Figure 4G–I). GSK-J4 administration effectively restored TJ protein expression: ZO-1 levels and claudin-5 levels were increased to 2.2 ± 0.4-fold and 2.1 ± 0.3-fold, respectively, compared with those in ICH mice (*p* = 0.017 and *p* = 0.014, respectively, two-tailed unpaired Student’s *t*-test, *n* = 3 per group; Figure 4G–I). Immunofluorescence staining of CD31^+^ cerebral microvessels further confirmed these findings: in vehicle-treated ICH mice, ZO-1 and claudin-5 exhibited discontinuous, fragmented expression along vessel walls, whereas GSK-J4 treatment preserved the linear, continuous localization of both TJ proteins (Figure 4J). Quantitative morphometric analysis confirmed these phenotypic changes: in the ICH group, the proportion of ZO-1^+^ and claudin-5^+^ areas within the total CD31^+^ vessel area was decreased by 75 ± 6% and by 87 ± 7%, respectively (both *p* < 0.001 vs. sham; *n* = 4 per group, one-way ANOVA for the initial analysis, and Sidak’s post hoc test for predefined pairwise comparisons, sham vs. ICH; Figure 4K,L), and GSK-J4 treatment significantly increased ZO-1^+^ signal to 5.9 ± 1.1-fold, and claudin-5^+^ signal to 7.5 ± 4.0-fold, respectively (*p* < 0.001 and *p* = 0.006, respectively; *n* = 4 per group, one-way ANOVA for the initial analysis, and Sidak’s post hoc test for predefined pairwise comparisons, ICH vs. ICH + GSK-J4; Figure 4K,L).

TEM provided high-resolution ultrastructural evidence of BBB protection by GSK-J4: sham mice displayed well-formed inter endothelial tight junctions (TJs), intact and continuous basement membranes (BM), and normal astrocyte endfeet (Figure 4M); In vehicle-treated ICH mice, however, structurally indistinct TJs, thin and discontinuous BM, and swollen astrocyte endfeet were observed (Figure 4M); GSK-J4 treatment significantly alleviated these ultrastructural abnormalities, with preserved visible TJs, a linear-shaped and continuous BM, as well as partially restored astrocyte endfeet (Figure 4M). Together, these results demonstrate that JMJD3 inhibition by GSK-J4 attenuates ICH-induced neuroinflammation and subsequently preserves BBB structural integrity by maintaining TJ protein expression and microvascular ultrastructure during the acute phase of injury.

### 3.4. JMJD3 Inhibition Accelerates Hematoma Resolution, Promotes Histological Recovery, and Improves Acute Sensorimotor Functions in ICH Mice

**Figure 5:**
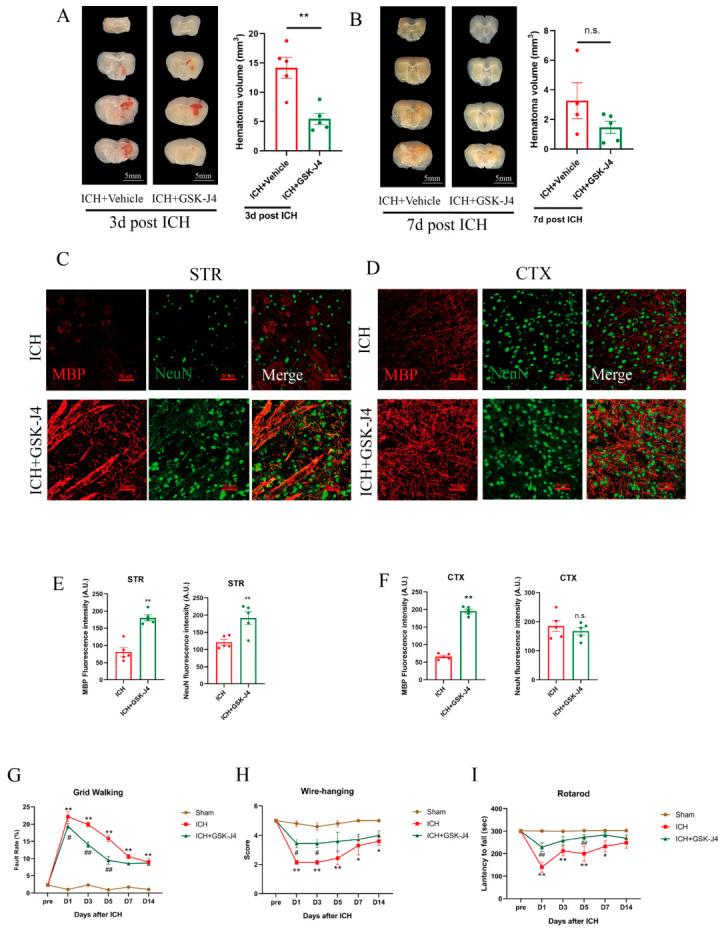
JMJD3 inhibition accelerates hematoma resolution, promotes white matter and neuronal recovery, and improves acute sensorimotor functions in ICH mouse model. (**A**,**B**) Effects of GSK-J4 on the hematoma volume are evaluated on serial coronal mouse brain sections collected at 3 days and at 7 days after ICH induction. The hematoma area (mm^2^) on each section was measured, and the hematoma volume (mm^3^) was determined by integrating the hematoma areas across all sections and multiplying by the section thickness (1 mm) using ImageJ. Quantitative analysis of hematoma volume in vehicle- and GSK-J4-treated ICH mice is shown at 3 days (**A**) and 7 days (**B**) post-ICH. Two-tailed unpaired Student’s *t*-test is used for analysis (ICH vs. ICH + GSK-J4). All values are presented as mean ± SEM from *n* = 4–5 animals per group. ** *p* < 0.01 vs. ICH group. n.s. not significant. Scale bars: 5 mm. (**C**,**D**) Effects of GSK-J4 on the histological recovery (white matter repair and neuronal survival) in ICH mice at 7 days after ICH induction are evaluated by immunofluorescence staining. Representative immunofluorescence images of myelin basic protein (MBP, a marker of white matter integrity, red) and neuron-specific nuclear protein (NeuN, a marker of viable neurons, green) staining in the perihematomal striatum (STR) (**C**) or in the ipsilateral cortex (CTX) (**D**) in sham, ICH, and ICH + GSK-J4 mice at 7 days post ICH. Scale bars: 50 μm. (**E**,**F**) Quantitative analysis of mean MBP and NeuN fluorescence intensity in STR (**E**) and in CTX (**F**) at 7 days in vehicle-treated and GSK-J4-treated ICH group. Two-tailed unpaired Student’s *t*-test is used for analysis (ICH vs. ICH + GSK-J4). All values are presented as mean ± SEM from *n* = 5 animals per group. ** *p* < 0.01, n.s. not significant vs. ICH group. (**G**–**I**) Effects of GSK-J4 on functional recovery are evaluated in ICH mice at 1, 3, 5, 7, and 14 days post-ICH. Sensorimotor function in sham, vehicle-treated, and GSK-J4-treated ICH mice is assessed by grid walking, wire-hanging, and rotarod test. (**G**) In the grid walking test, foot faults are recorded whenever a forepaw or a hindpaw slipped between the grid bars. The food fault rate was then calculated as the percentage of food faults relative to the total number of steps. (**H**) In the wire-hanging test, mice were hung by their forelimbs, and performance was scored based on a 0–5 scale. (**I**) In the rotarod test, the latency to fall was measured recorded as the time the mouse remained on the accelerating rotarod. Statistically, two-way ANOVA was used for initial analysis for the grid walking test and rotarod test, and *p*-values from predefined pairwise comparisons (sham vs. ICH, ICH vs. ICH + GSK-J4) were obtained following Sidak’s post hoc test. In the grid walking test and rotarod test, values are presented as mean ± SEM (*n* = 5 for sham, *n* = 7 for ICH and ICH + GSK-J4). For the wire hanging test, statistical analysis was performed using Wilcoxon Rank-Sum test (sham vs. ICH, ICH vs. ICH + GSK-J4), and values are presented as median with interquartile range (IQR) *(n* = 5 for sham, *n* = 7 for ICH and ICH + GSK-J4). * *p* < 0.05, ** *p* < 0.01 vs. sham group; # *p* < 0.05, ## *p* < 0.01 vs. ICH group.

We further extended the research by focusing on JMJD3 inhibition and its histological outcomes and functional recovery after ICH, evaluating hematoma resolution, white matter integrity, neuronal survival, and sensorimotor function. Hematoma volume analysis at three days post-ICH showed that GSK-J4 administration resulted in a 62 ± 8% shrinkage in hematoma volume compared to ICH mice receiving vehicle (*p* = 0.002, two-tailed unpaired Student’s *t*-test, *n* = 5 per group; Figure 5A), indicating accelerated hematoma resolution in the acute phase of injury. At seven days post-ICH, a continued decrease was observed in both groups, but the difference between them did not reach statistical significance (*p* = 0.164, two-tailed unpaired Student’s *t*-test, *n* = 4–5 per group; Figure 5B); This time-dependent trend suggests that JMJD3 inhibition exerts a robust early effect on hematoma clearance, which is a key determinant of secondary injury severity after ICH. To explore the underlying mechanism by which GSK-J4 accelerated hematoma clearance, we co-stained microglia with Iba-1 (microglia marker) and CD68 (phagocytic activity marker) in the ICH and ICH + GSK-J4 group 3 days post induction. The result showed that GSK-J4 treatment led to a 3.9 ± 0.9-fold (*p* = 0.001, two-tailed unpaired Student’s *t*-test, *n* = 5 per group) increase in the number of CD68^+^Iba-1^+^ phagocytic microglia in the peri-hematomal region on day 3. Therefore, we speculate that the phagocytosis of microglia may be involved in the accelerated hematoma clearance after GSK-J4 treatment in acute ICH (Supplementary Figure S3).

To assess histological recovery, we quantified the expression levels of myelin basic protein (MBP), a myelin sheath marker indicating white matter integrity, and neuron-specific nuclear protein (NeuN), a viable neuron marker indicating neuronal recovery, in the perihematomal striatum (STR) and ipsilateral cortex (CTX) at 7 days post-ICH. The results showed that in the perihematomal STR (a region of severe ICH-induced damage), GSK-J4 treatment significantly upregulated MBP and NeuN expression by 2.2 ± 0.4-fold and 1.6 ± 0.2-fold, respectively, compared to vehicle-treated ICH mice (both *p* < 0.001, two-tailed unpaired Student’s *t*-test, *n* = 5 per group; Figure 5C,E). In the ipsilateral CTX (a region of milder secondary damage), GSK-J4 treatment increased MBP expression to 3.0 ± 0.2-fold of the levels in the ICH group (*p* < 0.001, two-tailed unpaired Student’s *t*-test, *n* = 5 per group; Figure 5D,F), with no additional impact on NeuN expression (*p* = 0.484, two-tailed unpaired Student’s *t*-test, *n* = 5 per group; Figure 5D,F), which is likely attributable to minimal primary neuronal damage and spontaneous recovery in the cortical region. These data demonstrate that JMJD3 inhibition promotes white matter repair and neuronal survival in regions most severely affected by ICH.

To determine the functional consequences of these histological improvements, we also performed a battery of validated sensorimotor function tests (grid walking, wire-hanging, and rotarod) at baseline and on days 1, 3, 5, 7, and 14 post-ICH. Vehicle-treated ICH mice exhibited severe sensorimotor deficits from day 1 to day 5 post-injury versus sham mice in the grid-walking and rotarod tests (all *p* < 0.01, two-way ANOVA for the initial analysis, and Sidak’s post hoc test for predefined pairwise comparisons, sham vs. ICH), as well as in the wire-hanging test (all *p* < 0.01, Wilcoxon rank-sum test, sham vs. ICH) (*n* = 5 for sham, and *n* = 7 for ICH in all behavioral tests). GSK-J4 treatment significantly improved acute sensorimotor function in ICH mice: in the grid walking test, the foot fault rate was reduced by 19 ± 8% (day 1), 27 ± 7% (day 3), and 37 ± 5% (day 5) compared to vehicle-treated ICH mice (*p* = 0.034, *p* < 0.001, and *p* < 0.001, respectively; *n* = 7, two-way ANOVA for the initial analysis, and Sidak’s post hoc test from predefined pairwise comparisons, ICH vs. ICH + GSK-J4; Figure 5G); in the rotarod test, latency to fall was prolonged to 1.6 ± 0.3-fold (day 1) and 1.4 ± 0.2-fold (day 5) (*p* = 0.001 and *p* = 0.005, respectively; *n* = 7, two-way ANOVA for the initial analysis, and Sidak’s post hoc test for predefined pairwise comparisons, ICH vs. ICH + GSK-J4; Figure 5I); and in the wire-hanging test, the GSK-J4-treated group had a median score of 4 (IQR: 3–4) compared to 2 (IQR: 2–3) in the ICH group at both day 1 and day 3 (both *p* = 0.013; *n* = 7, Wilcoxon rank-sum test; Figure 5H). Notably, by day 7 post-ICH, spontaneous functional recovery occurred in all experimental groups, and no significant variations in sensorimotor abilities were observed between GSK-J4-treated and vehicle-treated ICH mice in grid-walking test and rotarod test (*p* = 0.209 and *p* = 0.452, respectively; *n* = 7, two-way ANOVA for the initial analysis, and Sidak’s post hoc test for predefined pairwise comparisons, ICH vs. ICH + GSK-J4), or the wire-hanging test (*p* = 0.918; *n* = 7, Wilcoxon rank-sum test, ICH vs. ICH + GSK-J4). These behavioral results indicate that JMJD3 inhibition by GSK-J4 exerts a robust protective effect on acute sensorimotor function during the critical early phase of ICH, a period associated with the most severe secondary injury and greatest potential for therapeutic intervention.

## 4. Discussion

Since the first histone demethylase (KDM) inhibitor, tranylcypromine (TCP), was discovered 20 years ago, KDM inhibitors have emerged as novel therapies for their neuroprotective effects. On the basis of their enzymatic activity, KDMs fall into two subfamilies: (1) flavin-containing lysine-specific demethylases (KDM1, also known as LSD), which can remove mono-and dimethyl groups; and (2) Jumonji C family protein KDMs (KDM2–8), capable of demethylating mono-, di-, and trimethyl groups [24]. Inhibitors of KDMs that show neuroprotective effects in the CNS include vafidemstat and modified TCP, which inhibit LSD1; ML324, which inhibits the KDM4 family; and GSK-J4, which inhibits the KDM6 family [51,52]. Vafidemstat has been reported to decrease the secretion level of cytokines IL12 and IL23 to enhance anti-inflammatory effect in experimental autoimmune encephalomyelitis [28]. Modified TCP has been reported to confer neuroprotective effects in TBI by alleviating redox imbalance [30]. KDM4 inhibitor ML324 has been reported to inhibit TNF-α-induced leukocyte transmigration in vitro [53].

However, in the context of hemorrhagic brain diseases, particularly ICH, histone deacetylase (HDAC) inhibitors have been extensively studied, whereas research on KDM inhibitors remains scarce [54,55]. The present study is dedicated to analyzing the role played by JMJD3 in astrocytes during ICH-induced neuroinflammation and BBB disruption. Our key findings are as follows: (1) JMJD3 was significantly upregulated in astrocytes following ICH (in vitro and in vivo) and correlated with neuroinflammatory signaling (IL-6/JAK/STAT3 signaling pathway); (2) JMJD3 inhibition via GSK-J4 reduced neuroinflammation (by suppressing iNOS, TNF-α, and MMP-9) and safeguarded BBB integrity (by stabilizing tight junction complexes and ultrastructure) post-ICH in the acute phase; (3) GSK-J4 administration enhanced histological and functional recovery (by reducing hematoma, preserving white matter and neural tissue, and improving acute sensorimotor function) post-ICH in the acute phase. These results identified astrocyte JMJD3 as a novel pharmacological target for attenuating early-stage cerebral injury following ICH.

### 4.1. Astrocytic JMJD3: A Common Epigenetic Regulator in CNS Injuries

The H3K27-preferential KDM6 family contains two members: UTX (also designated as KDM6A) and JMJD3 (KDM6B). As an epigenetic activator, JMJD3 is frequently upregulated in various pathological conditions, such as oxygen-glucose deprivation/reoxygenation (OGD/R) in vitro astrocyte model and cavitation-induced astrocyte injury model [39,40]. In addition to astrocytes, JMJD3 upregulation has also been observed in preclinical models of spinal cord injury (SCI), ischemic cerebral injury, and depression- and anxiety-like behavior [36,37,56,57,58].

In line with these observations, our hemin-induced astrocyte model exhibited a significant upregulation of *Jmjd3* gene, as validated by RNA-seq and qPCR, and we revealed *Jmjd3* expression correlated positively with the activation of IL-6/JAK/STAT3 signaling pathway. Moreover, pretreatment of hemin-induced astrocytes with GSK-J4 significantly reduced IL-6 secretion, functionally validating the pro-inflammatory role of astrocytic JMJD3 in ICH in vitro. Previous functional study in an OGD/R model has demonstrated that JMJD3 is involved in astrocytic apoptosis, as shown by the finding that dexmedetomidine exerts its anti-apoptotic effect by suppressing JMJD3 [39]. However, the direct transcriptional targets of JMJD3 in astrocytes have yet to be identified at the mechanistic level. In additional cell types (e.g., macrophages and vascular endothelial cells), chromatin immunoprecipitation (ChIP) experiments have shown that JMJD3 binds specifically to promoter sequences of genes encoding inflammatory factors (for example, IL-6, TNF-α) and removes the repressive H3K27me3 mark, thereby facilitating their expression [59,60]. Based on these findings and our functional data, we propose that JMJD3 may regulate the inflammatory process in astrocytes through similar mechanisms. One limitation of our RNA-seq results is that we did not validate additional targets, including other inflammatory cytokines, chemokines, and proteases. Future studies should employ comprehensive validation of RNA-seq targets, along with ChIP assays, particularly in primary astrocytes, to directly confirm the regulatory interactions.

Another crucial finding is that in our current work, JMJD3 was upregulated in astrocytes, but not in neurons, in the perihematomal regions three days after the induction of ICH mouse model. According to a previous report, neurons and astrocytes together account for 80–90% of the total cells in the striatum, with neurons comprising 50–60% and astrocytes 20–30% of the total cell population [61]. Our results highlight the upregulation of JMJD3 in astrocytes after ICH in vivo, suggesting that astrocytic JMJD3 may be the major source of JMJD3 upregulation in the perihematomal striatal tissue after ICH. A previous neuronal-related study has indicated that neuronal JMJD3 induction is linked to the expression of pro-survival genes following excitatory stimuli used for preconditioning, which are absent in our ICH model [62]. Therefore, we speculate that JMJD3 upregulation is highly context-dependent and, under ICH conditions, astrocytes are vital responding cells with elevated JMJD3 expression. We also observed an increase in JMJD3^+^Iba-1^+^ microglia in the perihematomal region, indicating multi-cellular involvement. However, given the predominant abundance of astrocytes in the striatum and their established role in BBB integrity and neuroinflammation, we focused our functional investigation on this cell type. The functional significance of JMJD3 upregulation in microglia during acute ICH remains to be fully elucidated. Taken together, our cell-based and animal data provide the first functional evidence that JMJD3 drives neuroinflammation in astrocytes under hemorrhagic conditions and support the broader application of astrocytic JMJD3 inhibition as a therapeutic approach against neuroinflammation in experimental models of CNS injury.

### 4.2. Therapeutic Values of GSK-J4 on Neuroinflammation and BBB Disruption After ICH

GSK-J4 is the prodrug of GSK-J1, with an ethyl ester group added to enhance cell permeability. It was originally developed by GlaxoSmithKline (Brentford, UK) and exhibits inhibitory activity against JMJD3 and UTX [33]. Although GSK-J4 has also been reported to suppress KDM5 family members at high concentrations, it is inactive against other Jumonji C demethylases and is therefore considered a selective KDM inhibitor [63]. Owing to its lipophilic properties, GSK-J4 can cross the BBB and shows therapeutic potential for CNS disorders. Its neuroprotective benefits have been validated using preclinical models of CNS injury. In an LPS-induced mouse model of anxiety-like behavior, administration of GSK-J4 prior to LPS exposure downregulates hippocampal expression of JMJD3 and subsequently reduces the secreted levels of IL-1β, TNF-α, and IL-6 [37]. In a rat spinal cord injury model with a dorsal surface contusion, GSK-J4 inhibited TJ protein degradation, thus protecting the impaired barrier function by downregulating JMJD3 and upregulating H3K27me3 [64].

In our ICH model, GSK-J4 treatment reduced neuroinflammation, as evidenced by suppressed expression of TNF-α, iNOS, and MMP-9 in the perihematomal striatal tissue 3 days after ICH. Our findings point to the conclusion that GSK-J4 exerts protective effects in ICH in vivo through its potent anti-inflammatory effects in the acute phase. The basis by which GSK-J4 inhibits JMJD3 has been established: Given 2-oxoglutarate (2-OG) is a key cofactor of JMJD3, therefore, GSK-J4 can act as a 2-OG analog, competitively binds to the active site of JMJD3 without mediating its demethylase activity, thereby inhibiting the enzyme [33]. Although off-target effects of GSK-J4 are possible, especially on UTX (KDM6A) and the KDM5 family. However, to our knowledge, the specific mechanism of GSK-J4 in inhibiting UTX has been rarely studied, and the result of UTX inhibition would also lead to H3K27me3 elevation; in addition, although the inhibitory effect of GSK-J4 on the KDM5 family may lead to H3K4me3, which results in gene activation, the literature has shown that GSK-J4/1 exhibits 5–10-fold lower inhibitory activity against KDM5 demethylases than against JMJD3 [33]. Therefore, we propose that the off-target effects of GSK-J4 can be neglected and that it can exert its neuroprotective effects in the ICH mouse model primarily through JMJD3 inhibition, resulting in global H3K27me3 elevation and suppression of pro-inflammatory pathways. This hypothesis is supported by a previous study confirming that daily i.p. injections of GSK-J4 for one week exerted anti-inflammatory effects in the hippocampus of LPS-challenged mice, via VGLL4 pathway [37]. Notably, the hippocampus is one of the brain regions with the highest abundance of astrocytes, where they constitute over 50% of the total cell population [61]. Given that astrocytes are also the primary cell type exhibiting upregulated JMJD3 expression in striatal tissue surrounding the hematoma, we speculate that GSK-J4 may act primarily on astrocytic JMJD3 to suppress inflammatory responses in astrocyte-enriched neural structures, like the hippocampus and striatum, in pathological conditions like ICH, thereby exerting a substantial neuroprotective effect at the organismal level by alleviating inflammation in these functionally important regions.

In our ICH model, GSK-J4 treatment preserved BBB structural integrity on day 3 after ICH within the perihematomal region, as evidenced by upregulated TJ protein abundance (e.g., ZO-1 and claudin-5) and restoration of brain microvascular ultrastructure. Evans Blue extravasation assay also provided evidence that therapeutic effect on GSK-J4 on reducing BBB permeability at the functional level. It is well-established that BBB disruption is an essential driver of ICH pathology. Although direct evidence for JMJD3 upregulation contributing to the structural damage of the BBB is lacking, JMJD3 inhibition has been shown to protect BBB integrity by suppressing certain mediators that degrade BBB components. In spinal cord injury models, for instance, rapamycin and imatinib, which inhibit the upstream signaling molecules mTOR and PDGFR, respectively, can indirectly downregulate JMJD3 expression and confer BBB protection by suppressing the pro-inflammatory ECM-degrading enzyme MMP-9 expression [57,64]. Recently, Park et al. utilized a rounded impactor tip to induce a controlled cortical impact (CCI) injury onto the murine cortex. Using this model, they demonstrated that the TRPM7 inhibitor carvacrol indirectly inhibits JMJD3 expression, which in turn suppresses MMPs and SUR1/TRPM4 expression in brain endothelial cells, there by attenuating BBB disruption [41]. These results are consistent with our finding that direct inhibition of JMJD3 by GSK-J4 also contributes to neuroprotection against acute ICH by attenuating neuroinflammation and subsequently protecting BBB integrity, highlighting the therapeutic potential of JMJD3 inhibition.

### 4.3. GSK-J4 Attenuates on Neuroinflammation-Mediated BBB Disruption After ICH: The Role of MMP-9

Matrix metalloproteinases (MMPs) are neutral proteases participating in cerebral pathology, among which MMP-9 is particularly upregulated in hemorrhagic brain and serves as a key inflammatory mediator that directly degrades the vascular basement membrane and extracellular matrix, as well as cellular junction components, leading to BBB disruption [14,65]. The temporal dynamics of MMP-9 expression have been evaluated post-ICH, demonstrating that levels are maximally upregulated at 24 h and remain elevated from 48 h to 7 days [65]. As anticipated, the result of our study showed that MMP-9 was significantly upregulated in perihematomal brain tissue at 3 days post-ICH, and GSK-J4 treatment attenuated this elevation, demonstrating that JMJD3 inhibition downregulates MMP-9 expression.

The cellular source of MMP-9 after CNS injury remains debated. Park et al. has reported that carvacrol prevents JMJD3 recruitment to the MMP-9 promoter in an OGD/R model of bEnd.3 cells, indicating that endothelial-derived MMP-9 may be a critical contributor to BBB breakdown in some contexts [41]. However, in hemorrhagic brain models, some evidence has shown that astrocytes represent the main source of MMP-9 expression. For instance, in collagenase-induced ICH models, MMP-9 was found to be predominantly colocalized with reactive astrocytes, with weaker expression in microglia and minimal colocalization with endothelial cells post-ICH [66,67]. Similarly, in a subarachnoid hemorrhage (SAH) model, MMP-9 expression in reactive astrocytes rose steadily over the first week after ICH, with the highest expression observed on day 3, whereas other cell types exhibited negligible upregulation [68]. As proposed by Feng et al., this may be because in ICH or SAH, astrocytes and their perivascular endfeet are directly exposed to released blood components immediately after vessel rupture, whereas endothelial cells, located in the inner layer of the BBB, are affected later [68]. This is mechanistically plausible, as blood constituents, including thrombin, can directly stimulate the expression of MMP-9 in astrocytes via activation of protease-activated receptors (PARs) [23]. Although we did not directly evaluate MMP-9 co-localization with GFAP in our ICH model, and cannot exclude contributions from other cell types including microglia and endothelial cells, existing studies have established that MMP-9 is upregulated in astrocytes after hemorrhagic brain injury [67,68]. Based on this evidence, we propose that GSK-J4 preserves BBB integrity after ICH at least in partly by inhibiting MMP-9 secretion, with astrocytes likely serving as a major effector cell type. Mechanistically, we propose that in astrocytes, JMJD3 is recruited to the MMP-9 gene promoter, where it removes the repressive H3K27me3 mark and thereby promotes MMP-9 transcription. GSK-J4 treatment inhibits this recruitment, preventing JMJD3 from binding to the MMP-9promoter and subsequently suppressing MMP-9 expression. This mechanism aligns with the reported role of JMJD3 inhibition in reducing MMP-9 expression in endothelial cells under OGD/R conditions [41]. Future studies using ChIP assays will be needed to directly verify the binding of JMJD3 to the MMP-9 promoter in astrocytes. Notably, other neuroinflammatory mediators (e.g., TNF-α, IL-1β, ROS) have also been shown to promote astrocytic MMP-9 secretion, indicating that JMJD3 may influence BBB disruption through cumulative effects of multiple neuroinflammatory pathways [69,70,71]. Combining our in vivo data, we demonstrate that GSK-J4 directly reduces MMP-9 expression to protect BBB integrity. At the same time, reduced neuroinflammation likely contributes secondarily to BBB preservation. Thus, both direct (MMP-9-mediated) and indirect (neuroinflammation-mediated) mechanisms act in concert. In summary, the present evidence suggests that MMP-9 represents a critical effector through which GSK-J4, via JMJD3 inhibition, attenuates BBB disruption, although the specific cellular source of MMP-9 requires further investigation.

### 4.4. GSK-J4 Promotes Histological and Neurological Recovery in ICH: By Accelerating Hematoma Resolution in Acute Phase

The effects of JMJD3 inhibition at the histological and functional level are further supported by our data indicating that GSK-J4 accelerated hematoma resolution as early as day 3 after ICH. Hematoma resolution is a multifactorial process driven in part by dampening of overactivation of both brain-resident and circulating immune cells, together with reduced secretion of pro-inflammatory mediators and chemokines [14]. One possible mechanism for GSK-J4 on hematoma resolution in the acute phase of ICH is that GSK-J4 may enhance the early phagocytosis of microglia. Microglia are one of the primary phagocytic cells in ICH, with CD68 serving as a marker of active phagocytosis. However, CD68 expression remains at low levels within the first 3 days after ICH [72,73]. In contrast to this low basal level, our results indicated that GSK-J4 treatment increased the number of CD68-positive phagocytic microglia at day 3. Future studies are warranted to dissect the causal relationship between JMJD3 inhibition and early enhanced microglial phagocytosis, as well as to explore other potential mechanisms involved in hematoma resolution, such as erythrocyte lysis, heme metabolism, and iron handling. Our results also demonstrated that GSK-J4-treated mice exhibited improved white matter integrity and neuronal recovery, along with better sensorimotor function in the early phase of ICH. We speculated that these structural and functional improvements may result from early BBB reconstruction by GSK-J4 during the acute phase of ICH, subsequently providing a favorable environment for brain tissue repair [12].

In addition, according to our findings, hematoma volumes at 7 days post-ICH did not significantly differ between the groups with or without GSK-J4 treatment. This lack of difference may be explained by the endogenous recovery mechanisms of ICH seen from day 3, when the inflammatory response begins to change into a reparative phase to accelerate hematoma absorption and promote neuronal healing [74]. While the role of astrocytic JMJD3 in the reparative phase remains to be explored, microglia may emerge as key players in this stage, which may undergo a phenotypic shift and facilitate the clearance of extravasated erythrocytes in the brain and the generation of protective factors that counteract neuroinflammation and tissue, including IL-4, IL-10, TGF-β, and BDNF [75]. Tao et al. demonstrated that dehydroepiandrosterone (DHEA) exerts anti-inflammatory effects in hemin-induced BV-2 microglial cells, whereas GSK-J4 abolishes these beneficial effects by inhibiting M2 microglial activation, a phenotype associated with neuroprotection, leading to decreased transcription of anti-inflammatory genes [76]. The dual role of JMJD3 in inflammation complicates the therapeutic use of GSK-J4 in ICH [77]. Nevertheless, our data suggest that, at least in the acute phase of ICH, JMJD3 inhibition protects the BBB and alleviates injury by targeting astrocytes, as further evidenced by improved histological and functional recovery within 7 days after onset. And the long-term effects of GSK-J4 on functional recovery after ICH need to be thoroughly explored in the future. We speculate that the therapeutic efficacy of GSK-J4 in ICH is likely context- and time-dependent. Therefore, future studies should consider the timing of administration and the specific cell types involved when evaluating its therapeutic potential [77]. In summary, our study demonstrates that GSK-J4 confers histological and functional neuroprotection against acute ICH within the first 7 days post-injury, likely by preserving BBB integrity through multiple mechanisms. Further studies are warranted to investigate its effects in the subacute and chronic phases.

### 4.5. Translational Implications and Limitations

Our findings have important translational implications. GSK-J4 is a well-characterized, brain-penetrant JMJD3 inhibitor [32,78] with high animal tolerability. A previous study showed that immunodeficient mice that received 50 mg/kg GSK-J4 via i.p. injection for 3 consecutive weeks exhibited no signs of toxicity or weight reduction within the liver, kidney, or intestine [79]. In addition, GSK-J4 concentrations as high as 10 μM did not compromise cell growth or viability of lung fibroblasts [80]. The doses of GSK-J4 used in our animal and cellular experiments were lower than those mentioned above. Future studies are warranted to further evaluate the safety profile of GSK-J4 in vivo.

This study has several limitations: (1) We used a cell line (C8-D1A) for in vitro experiments because it is widely used. However, primary astrocyte cultures may better recapitulate in vivo astrocyte behavior, and future studies using primary mouse astrocytes are warranted to validate our findings. (2) We admit that the direct transcriptional regulation of MMP-9 by inhibiting astrocytic JMJD3 after ICH has not been directly proven, and future studies should confirm this by ChIP assays or astrocyte-specific JMJD3 knockout studies. (3) We mainly focused on acute outcomes (3 days post-ICH) for JMJD3 inhibition after ICH, and the role of JMJD3 inhibition in the subacute reparative phase of ICH and its influence on chronic sequelae remain to be elucidated. (e.g., cognitive impairment, post-stroke epilepsy). (4) We did not explore the role of JMJD3 in microglia, endothelial cells, and pericytes, or their involvement in ICH-induced neuroinflammation. A comprehensive study of JMJD3 in different glial cells (astrocytes and microglia) and vascular cells (endothelial cells and pericytes) is warranted in future ICH studies. (5) We acknowledge that the sample size for in vivo Western blot quantification (*n* = 3 per group) is relatively limited. Although the differences were statistically significant and consistent with our histological and behavioral data, a larger sample size would enhance statistical power. (6) Another limitation is the prophylactic dosing regimen, in which GSK-J4 was administered starting 3 days before ICH (Days −3, −1, 1, and 3). Although this pre-treatment approach was used to maximize JMJD3 inhibition and establish proof-of-concept for its role in ICH pathophysiology, it does not reflect a clinically relevant therapeutic time window. Notably, our in vitro data showed that GSK-J4 added simultaneously with hemin also reduced IL-6 secretion, suggesting that post-ICH administration may be effective. Future studies should evaluate post-ICH administration of GSK-J4 or more selective JMJD3 inhibitors to improve translational relevance.

## 5. Conclusions

In conclusion, astrocytic JMJD3 is significantly upregulated following ICH and drives the induction of neuroinflammation in the perihematomal region. Pharmacological inhibition of JMJD3 by GSK-J4 exerts robust neuroprotective effects in a murine ICH model by attenuating neuroinflammation and subsequent blood–brain barrier disruption, at least in part, through the suppression of astrocyte-derived pro-inflammatory signaling and MMP-9 expression. Collectively, our findings identify astrocytic JMJD3 as a novel epigenetic target for early therapeutic intervention in acute ICH, which merits further preclinical and translational investigation to validate its clinical potential.

## Figures and Tables

**Table 1:** Primer sequences for quantitative Real-Time polymerase chain reaction (qRT-PCR).

Gene Name	Primer Forward (5′ to 3′)	Primer Reverse (3′ to 5′)
*Jmjd* *3*	ACCCGACCTCTTACATCCCC	GGAGCAGGTTTGAGCACCAT
*Jmjd* *1* *a*	TCAGAGCTAGAGTCGGCTGG	CCAACTTTCTCCGAGCGTGA
*Jmjd* *1* *c*	CGACTGGGACACGGGTCTA	GTCTGGCTAGGGTCCTTCCT
*Jmjd* *2* *b*	CCAATGCTGTACGTGTTGCC	TGACGCCGGCTCTTTTTGAT
*Jmjd* *5*	CATCTGTGGACTCTGTGGGAA	TCCTGACAGACTGACTCTGAT
*Jmjd* *6*	CATCACTGCCACCCAGAAGACTG	AGTCCATTTCTCCTGTGCGG
*Gapdh*	CATCACTGCCACCCAGAAGACTG	ATGCCAGTGAGCTTCCCGTTCAG

## Data Availability

The RNA sequencing data analyzed in the current study are available in the Genome Sequence Archive (GSA) at the China National Center for Bioinformation under accession number CRA039150 (https://ngdc.cncb.ac.cn/gsa/browse/CRA039150) (accessed on 26 February 2026). The raw data supporting the conclusions of this article will be made available by the authors on request.

## Supplementary Materials

The following supporting information can be downloaded at: https://www.mdpi.com/article/10.3390/brainsci16050454/s1: Supplementary Figure S1. Immunofluorescence staining of JMJD3 expression in Iba-1-positive microglia in ICH mouse model and in sham mice at three days post-ICH; Supplementary Excel File S1. Raw cell counting data for JMJD3^+^Iba-1^+^ double-positive microglia, corresponding to Supplementary Figure S1; Supplementary Figure S2. Original uncropped Western blot images corresponding to Figure 4A,G; Supplementary Figure S3. Immunofluorescence staining of CD68 expression in Iba-1-positive microglia in ICH mice at three days post-induction, with or without GSK-J4 treatment; Supplementary Excel File S2. Raw cell counting data for CD68^+^Iba-1^+^ double-positive microglia, corresponding to Supplementary Figure S3.

## Abbreviations

The following abbreviations are used in this manuscript:

JMJD3	Jumonji C domain-containing protein 3
ICH	intracerebral hemorrhage
BBB	blood–brain barrier
PAR	protease-activated receptor
DAMP	damage-associated molecular pattern
IL-1β	interleukin-1β
TNF-α	tumor necrosis factor-α
C1q	complement 1q
IL-15	interleukin-15
CCL2	C-C motif chemokine ligand 2
CCL5	C-C motif chemokine ligand 5
NO	nitric oxide
iNOS	inducible nitric oxide synthase
MMP-9	matrix metalloproteinase-9
KMT	histone lysine(K) methyltransferase
KDM	histone lysine(K) demethylase
H3K27me3	histone H3 at lysine(K) 27 trimethylation
CNS	central nervous system
TBI	traumatic brain injury
SCI	spinal cord injury
SAH	subarachnoid hemorrhage
LPS	Lipopolysaccharide
DMSO	dimethyl sulfoxide
IL-6	interleukin-6
DEG	differentially expressed gene
GSEA	gene set enrichment analysis
GSVA	gene set variation analysis
ELISA	enzyme-linked immunosorbent assay
RT-qPCR	quantitative reverse transcription polymerase chain reaction
WB	Western blot
IF	immunofluorescence
TEM	transmission electron microscopy
SEM	standard error of the mean
ChIP	chromatin immunoprecipitation
GFAP	glial fibrillary acidic protein
NeuN	neuron-specific nuclear protein
CD31	platelet endothelial cell adhesion molecule 1
ZO-1	zonula occludens protein-1
MBP	myelin basic protein
TJ	tight junction
BM	basement membrane
ROI	region of interest
MFI	mean fluorescence intensity
OGD/R	oxygen-glucose deprivation/reoxygenation
Gapdh	glyceraldehyde-3-phosphate dehydrogenase
WMI	white matter injury
