# Supplementary material for: Inhibition of Astrocytic JMJD3 Attenuates Neuroinflammation-Mediated Blood–Brain Barrier Disruption and Improves Functional Recovery After Intracerebral Hemorrhage in Mice
Source: Brain Sci. 2026 Apr 24;16(5):454. doi: 10.3390/brainsci16050454 (PMC13204954; doi:10.3390/brainsci16050454)
Supplement: Supplementary file 1 [file brainsci-16-00454-s001.zip › brainsci-4238353-supplementary/Supplementary Materials/Supplementary Figure S1.pdf]

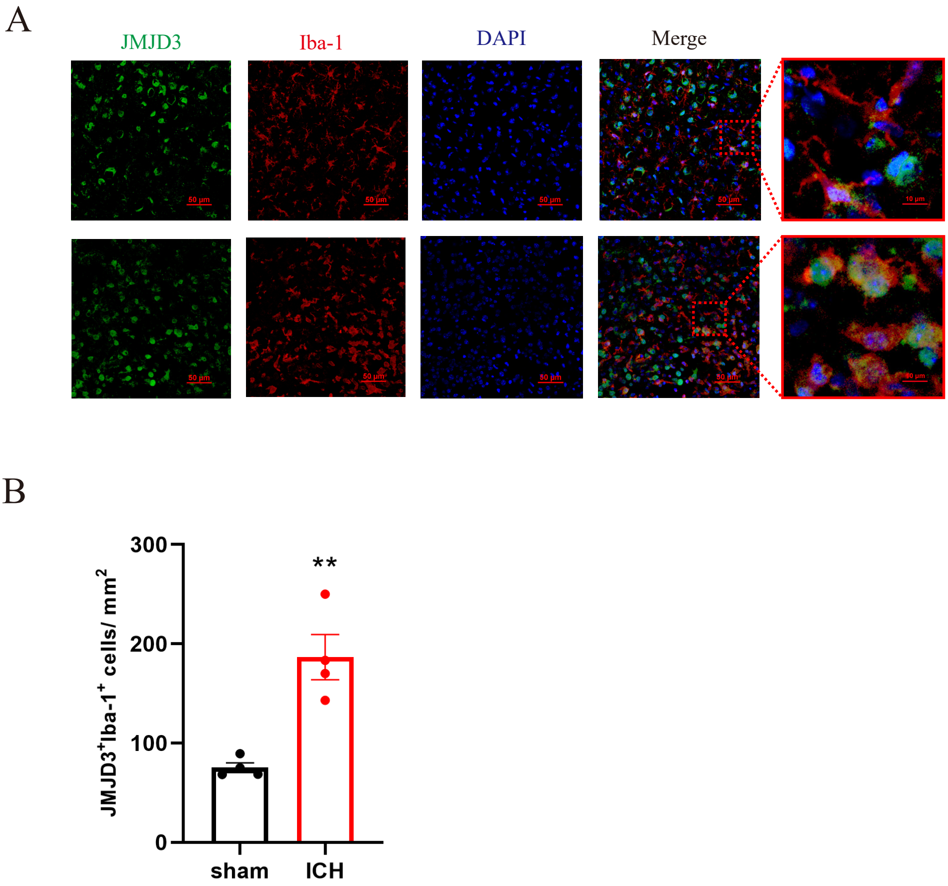

Supplementary Figure S1. Immunofluorescence staining of JMJD3 expression in Iba-1-postive microglia in ICH mouse model and in sham mice at three days post-ICH. (A) Representative immunofluorescence images showing JMJD3 (green) and Iba-1 (microglia marker, red) co-staining in the perihematomal regions of ICH mice and in sham mice. Enlarged views of the boxed regions reveal representative $JMJD3^{+}Iba\text{-}1^{+}$ double-positive microglia. Scale bars: 50 μm (insets: 10 μm). (B) Quantitative analysis of the number of double-positive microglia per mm^2. Values were averaged from three ROIs per mouse. Two-tailed unpaired Student's t-test is used for analysis (sham vs. ICH). All values are presented as mean ± SEM from n = 4 animals per group. $^{**}p < 0.01$.
