# Supplementary material for: Inhibition of Astrocytic JMJD3 Attenuates Neuroinflammation-Mediated Blood–Brain Barrier Disruption and Improves Functional Recovery After Intracerebral Hemorrhage in Mice
Source: Brain Sci. 2026 Apr 24;16(5):454. doi: 10.3390/brainsci16050454 (PMC13204954; doi:10.3390/brainsci16050454)
Supplement: Supplementary file 1 [file brainsci-16-00454-s001.zip › brainsci-4238353-supplementary/Supplementary Materials/Supplementary Figure S2.pdf]

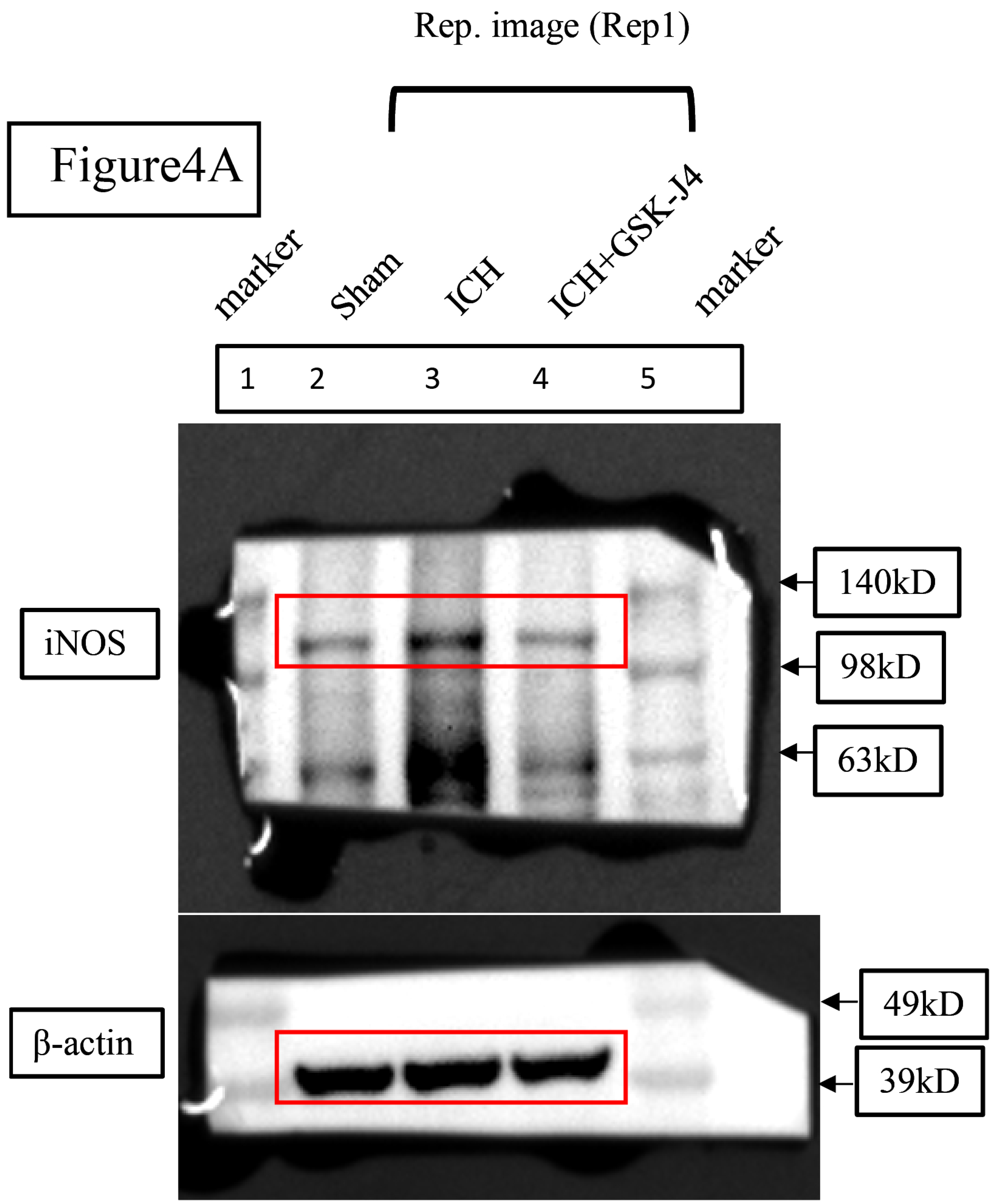
Rep. image (Rep1)
Figure4A
marker
Sham
ICH
ICH+GSK-J4
marker
1
2
3
4
5
iNOS
140kD
98kD
63kD
β-actin
49kD
39kD

Figure4A

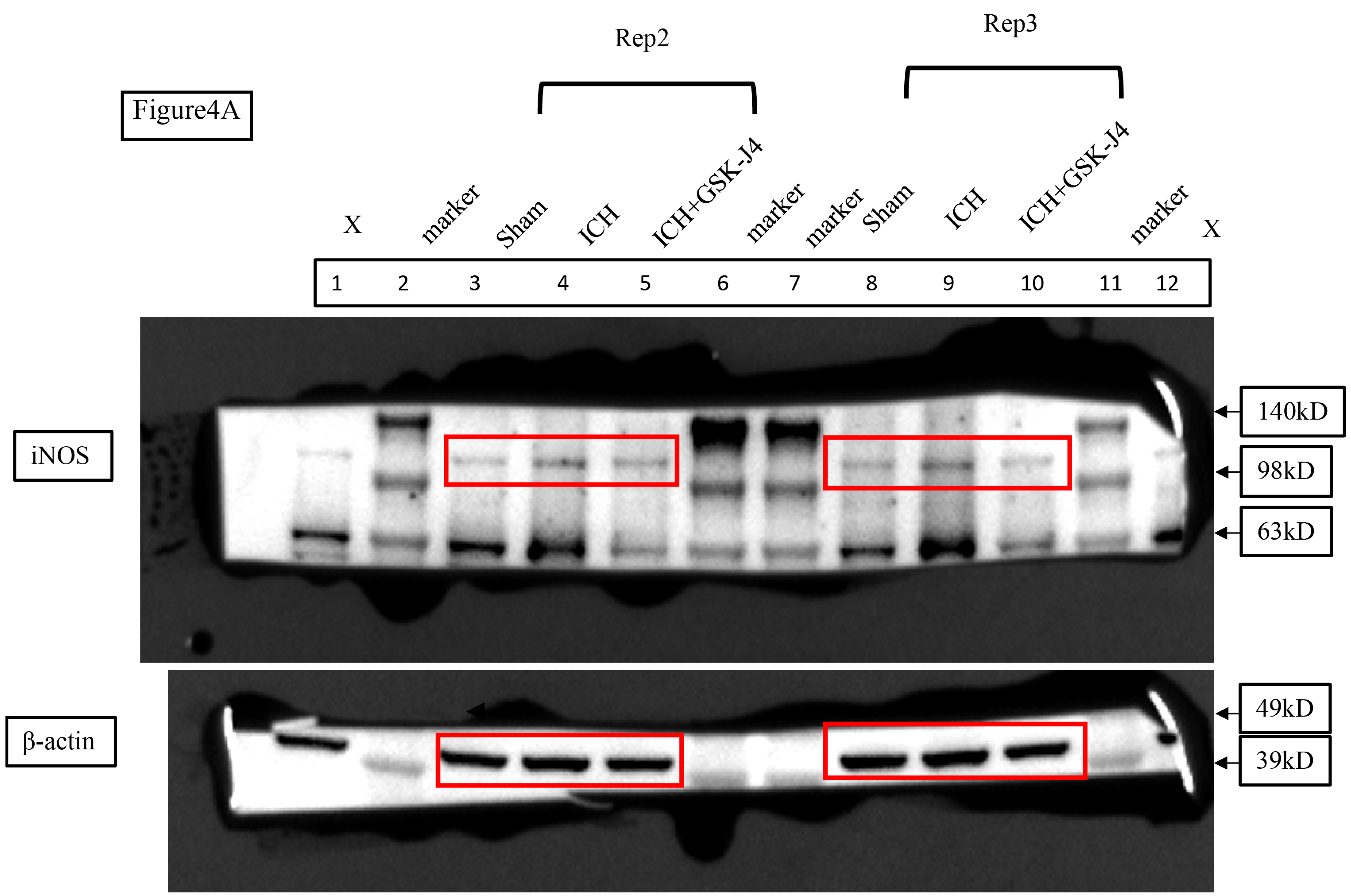

Rep2
Rep3
X
marker
Sham
ICH
ICH+GSK-J4
marker
marker
Sham
ICH
ICH+GSK-J4
marker
X
1
2
3
4
5
6
7
8
9
10
11
12
iNOS
β-actin
140kD
98kD
63kD
49kD
39kD

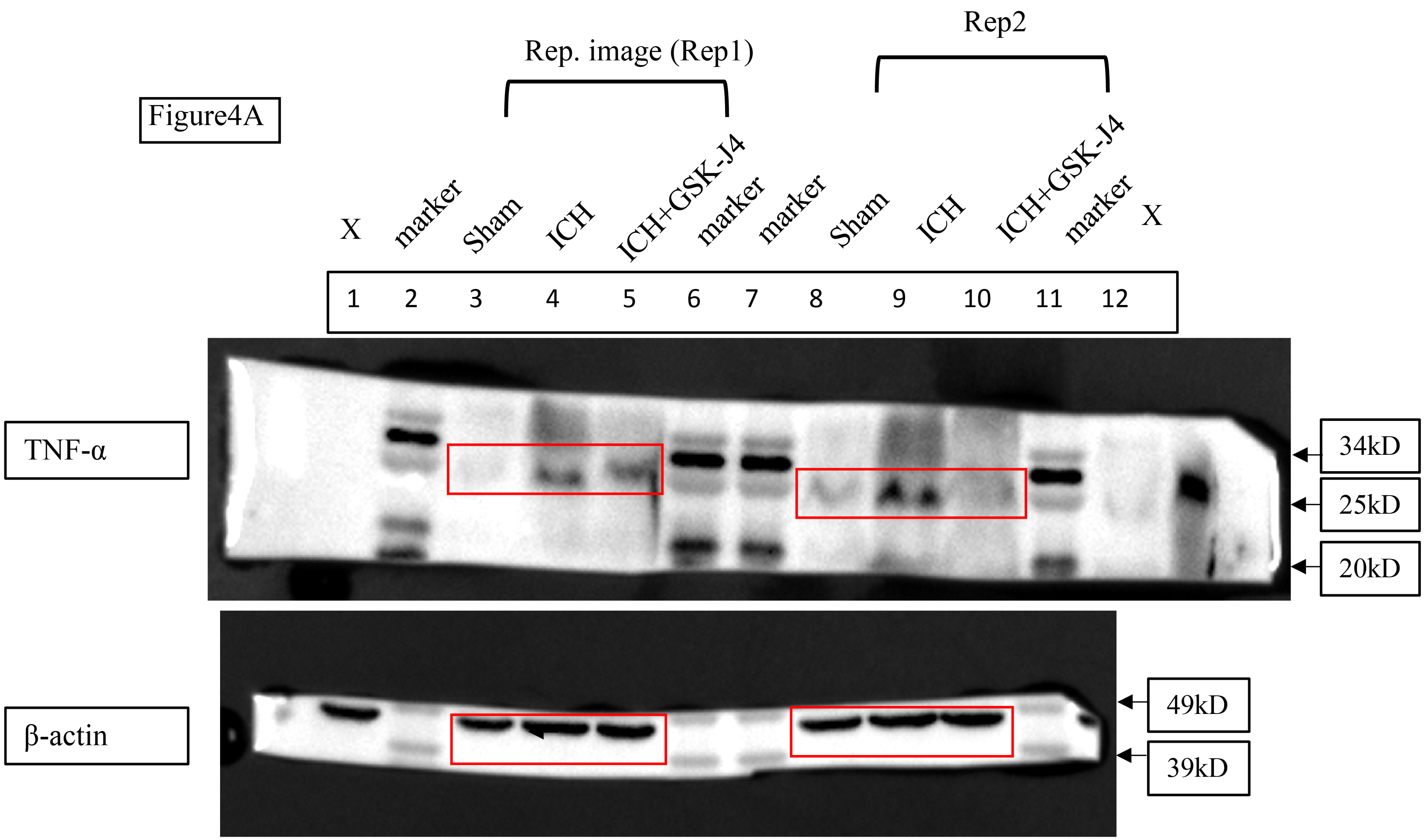
Figure4A
Rep. image (Rep1)
Rep2
X
marker
Sham
ICH
ICH+GSK-J4
marker
marker
Sham
ICH
ICH+GSK-J4
marker
X
1
2
3
4
5
6
7
8
9
10
11
12
TNF-α
β-actin
34kD
25kD
20kD
49kD
39kD

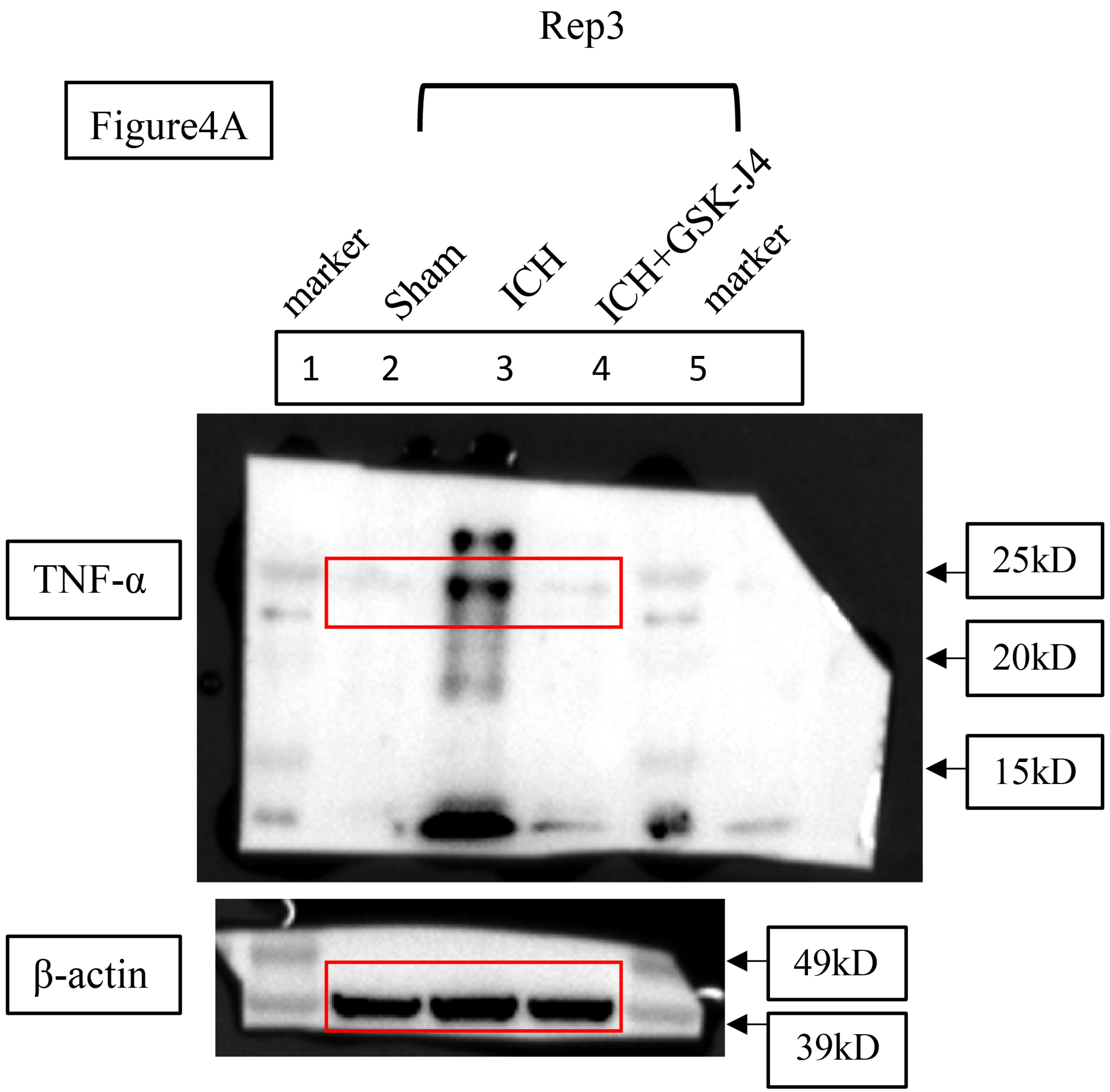

Figure4A
Rep3
marker
Sham
ICH
ICH+GSK-J4
marker
1 2 3 4 5
TNF-α
25kD
20kD
15kD
β-actin
49kD
39kD

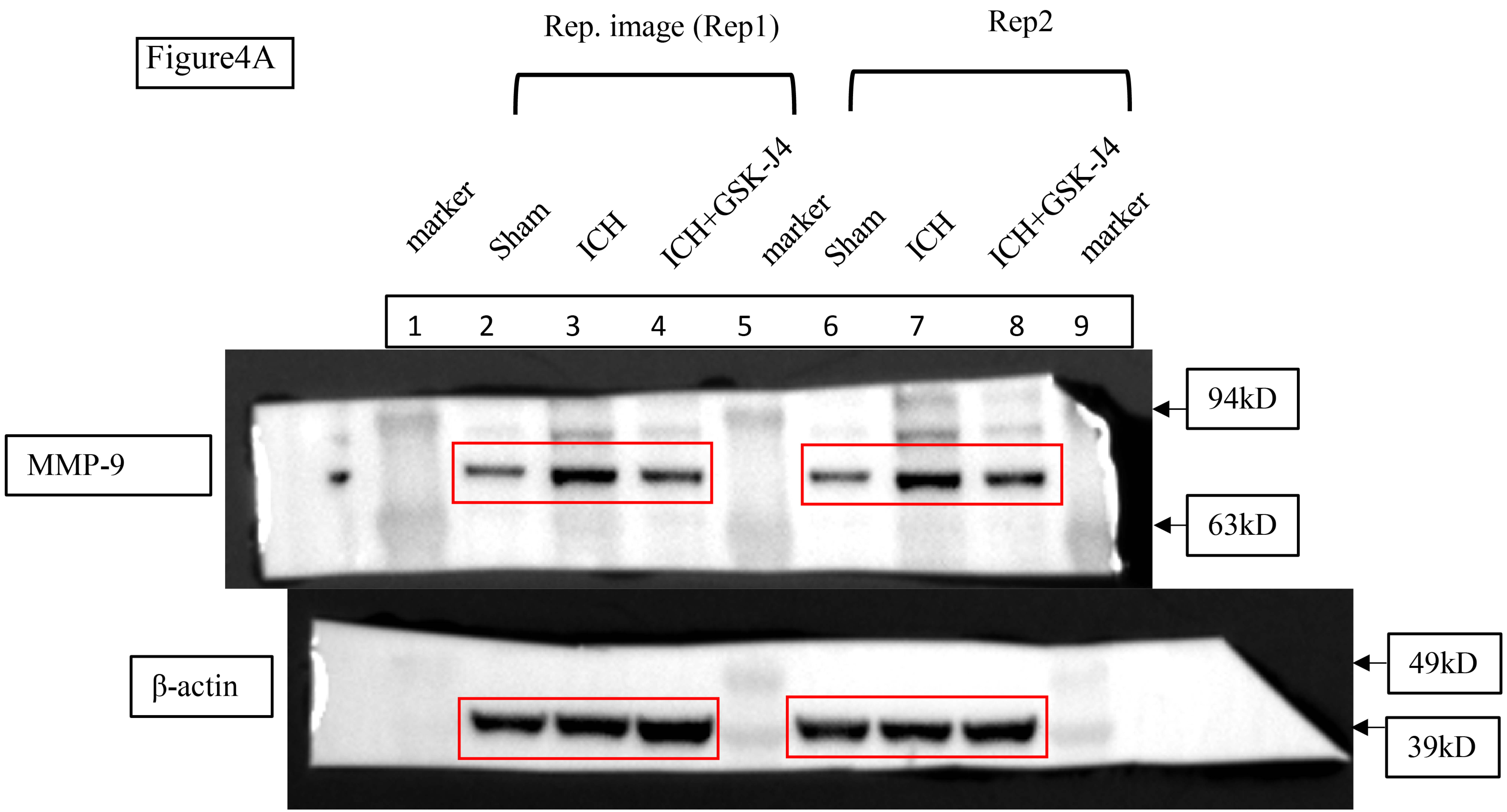
Figure4A
Rep. image (Rep1)
Rep2
marker
Sham
ICH
ICH+GSK-J4
marker
Sham
ICH
ICH+GSK-J4
marker
1 2 3 4 5 6 7 8 9
MMP-9
94kD
63kD
β-actin
49kD
39kD

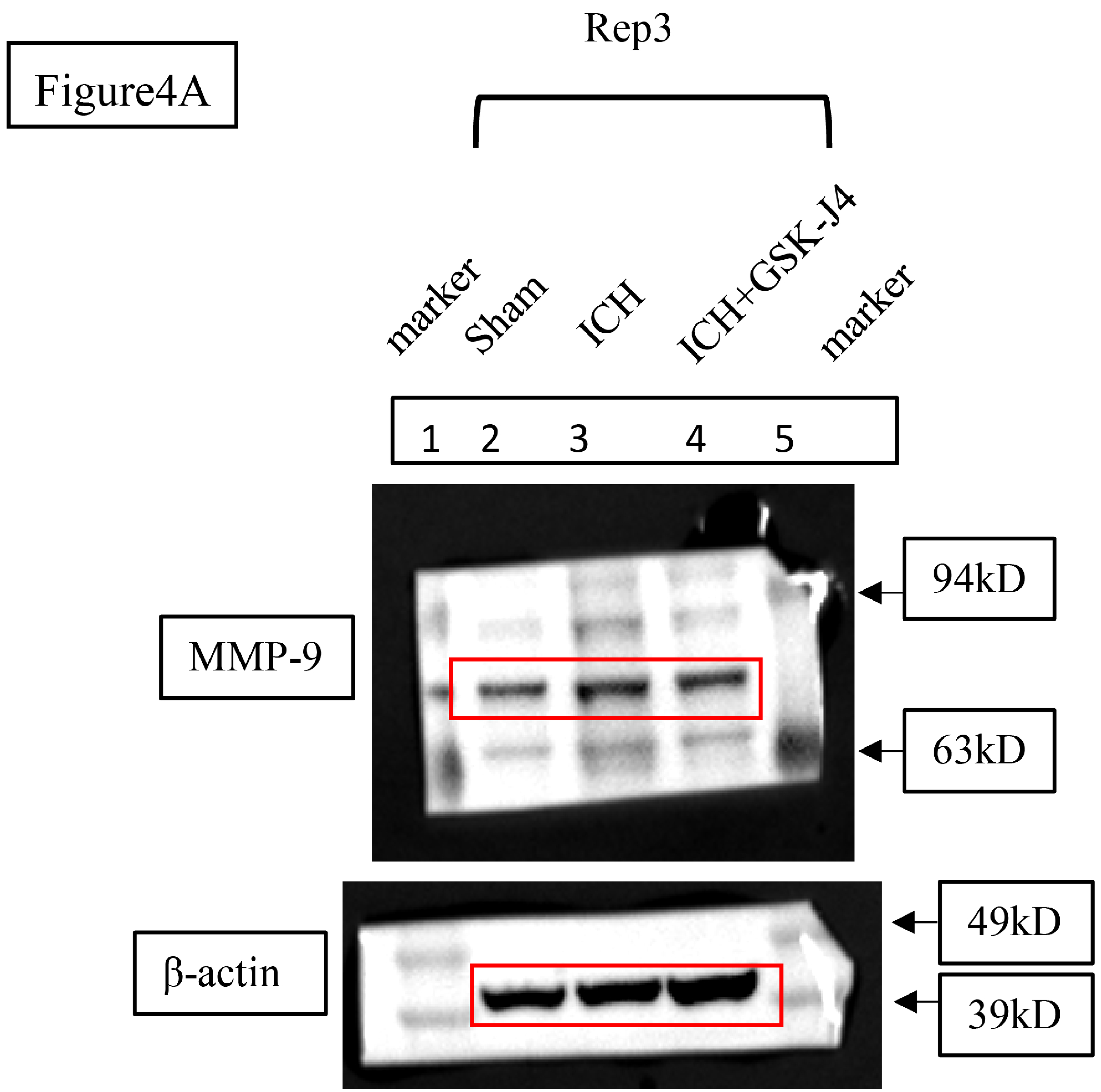

Figure4A
Rep3
marker
Sham
ICH
ICH+GSK-J4
marker
1 2 3 4 5
MMP-9
94kD
63kD
β-actin
49kD
39kD

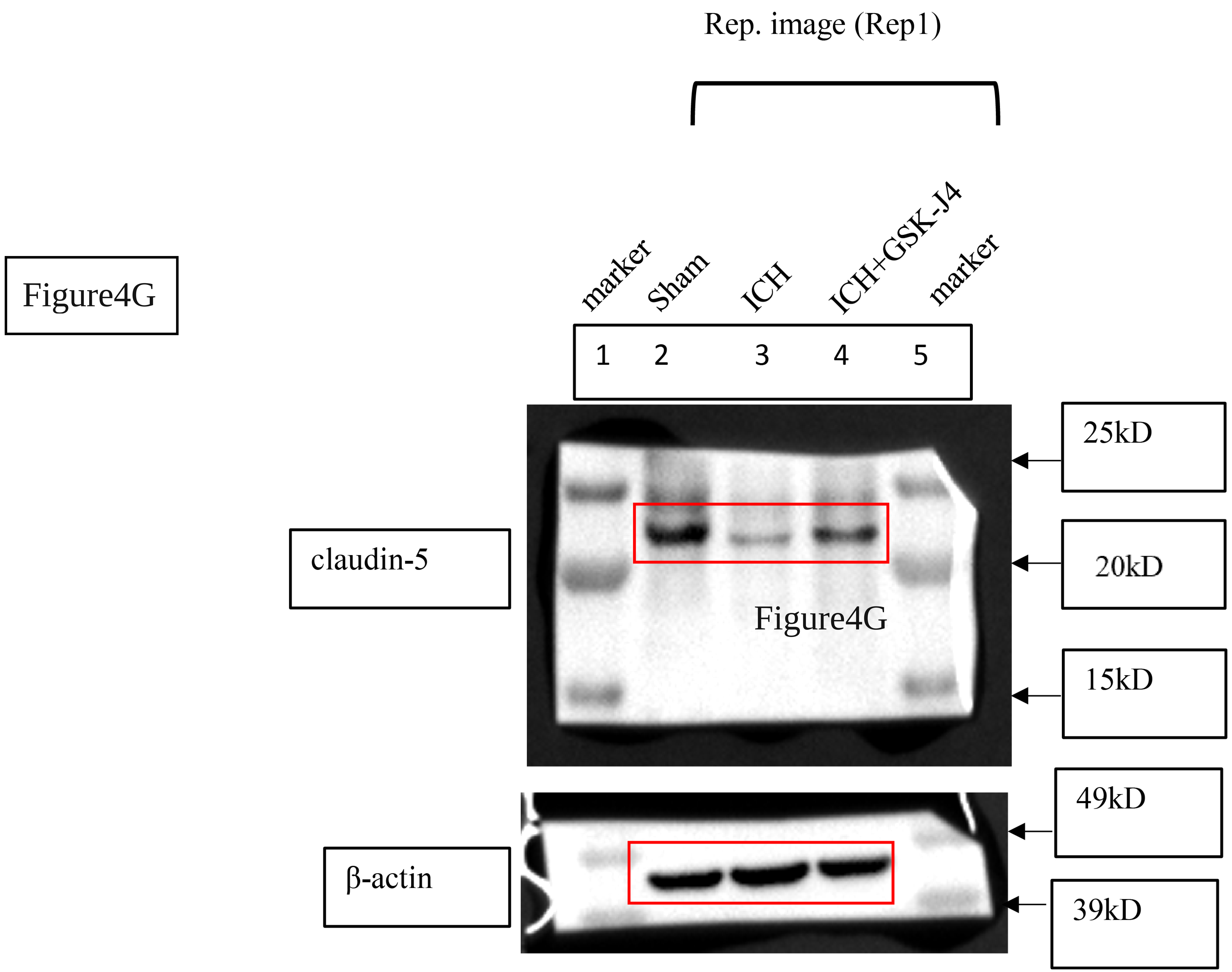

Rep. image (Rep1)
Figure4G
marker
Sham
ICH
ICH+GSK-J4
marker
1
2
3
4
5
claudin-5
Figure4G
25kD
20kD
15kD
49kD
39kD
β-actin

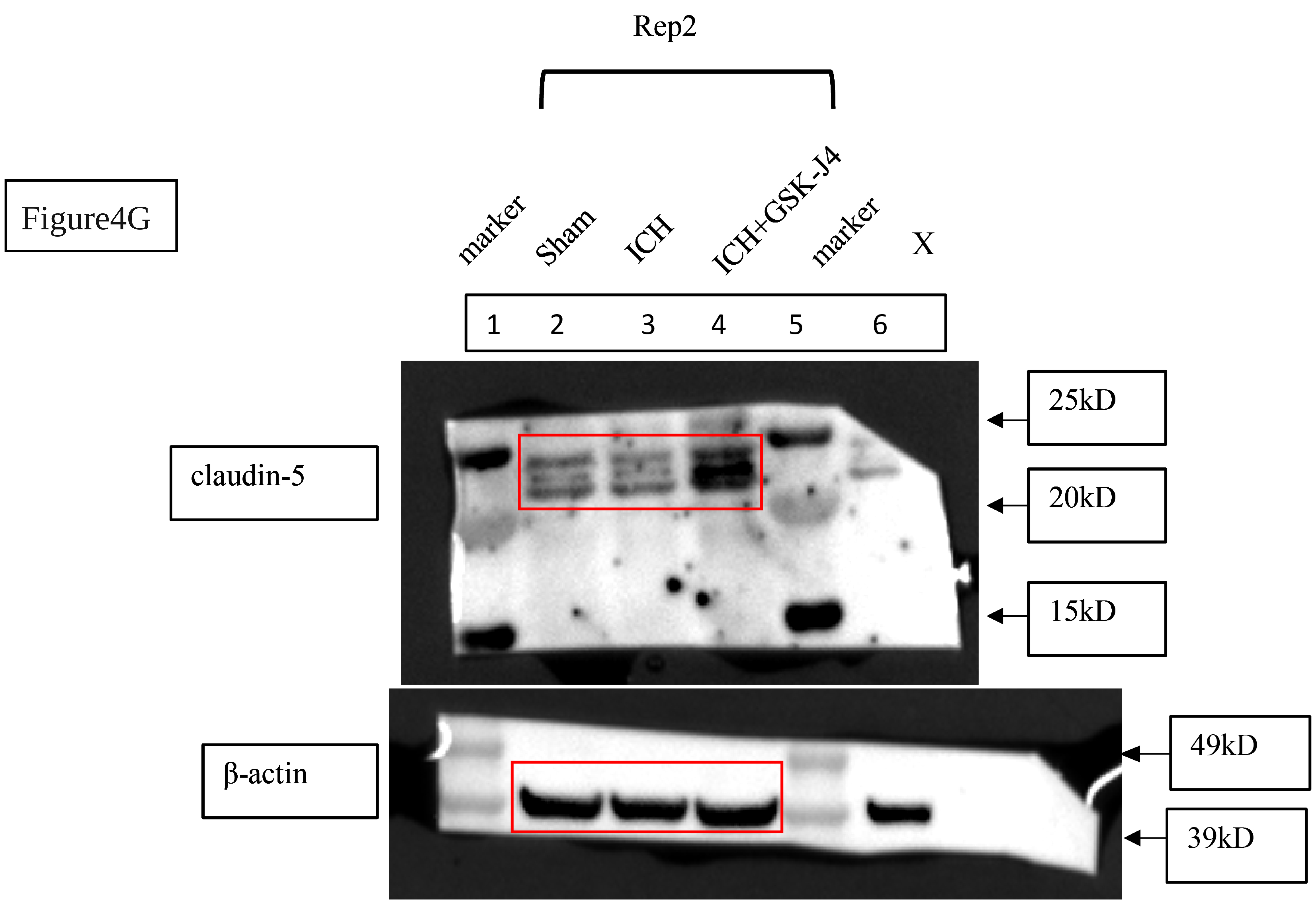
Rep2
Figure4G
marker
Sham
ICH
ICH+GSK-J4
marker
X
1
2
3
4
5
6
claudin-5
25kD
20kD
15kD
β-actin
49kD
39kD

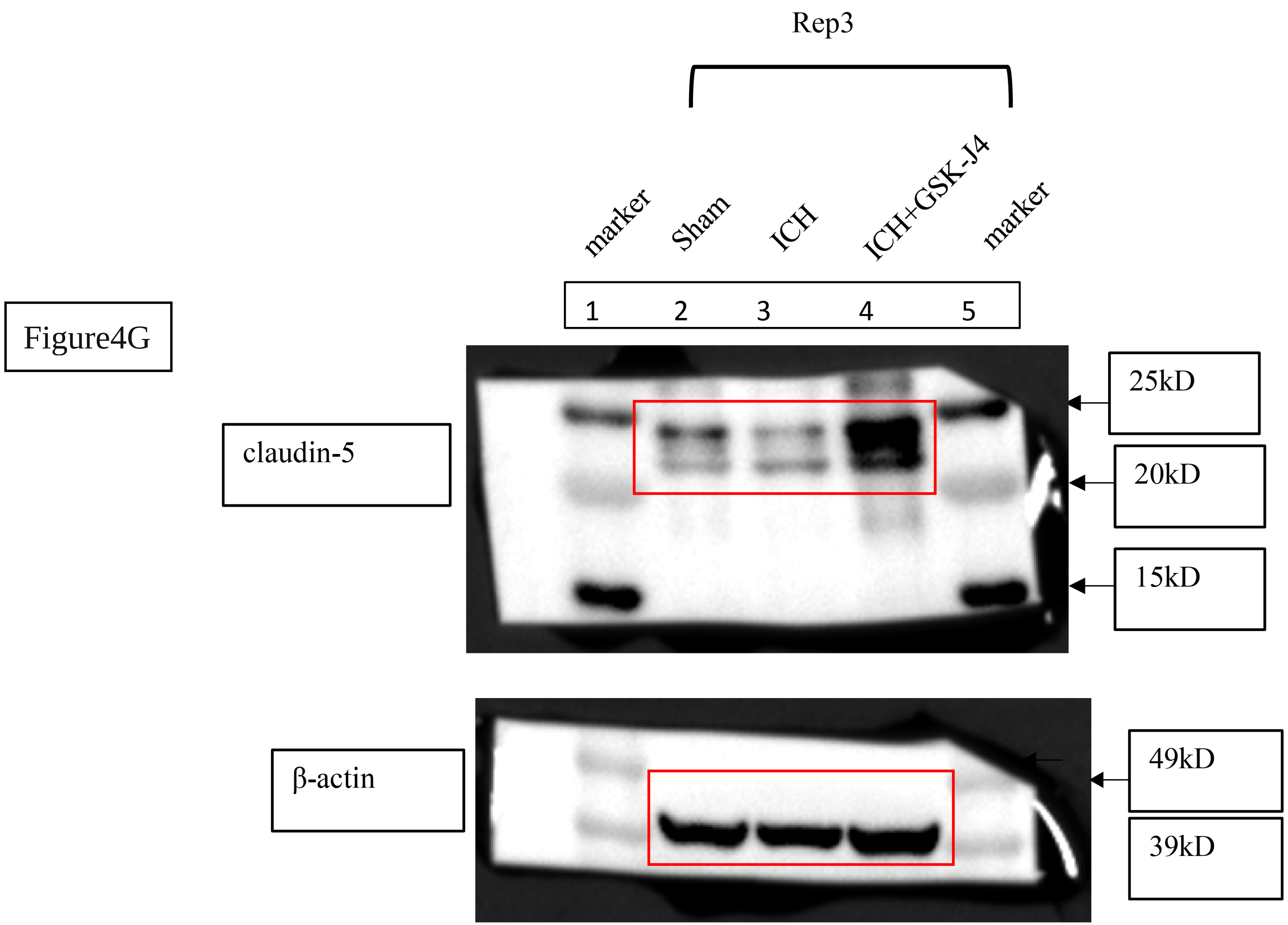

Figure4G
Rep3
marker
Sham
ICH
ICH+GSK-J4
marker
1
2
3
4
5
claudin-5
25kD
20kD
15kD
β-actin
49kD
39kD

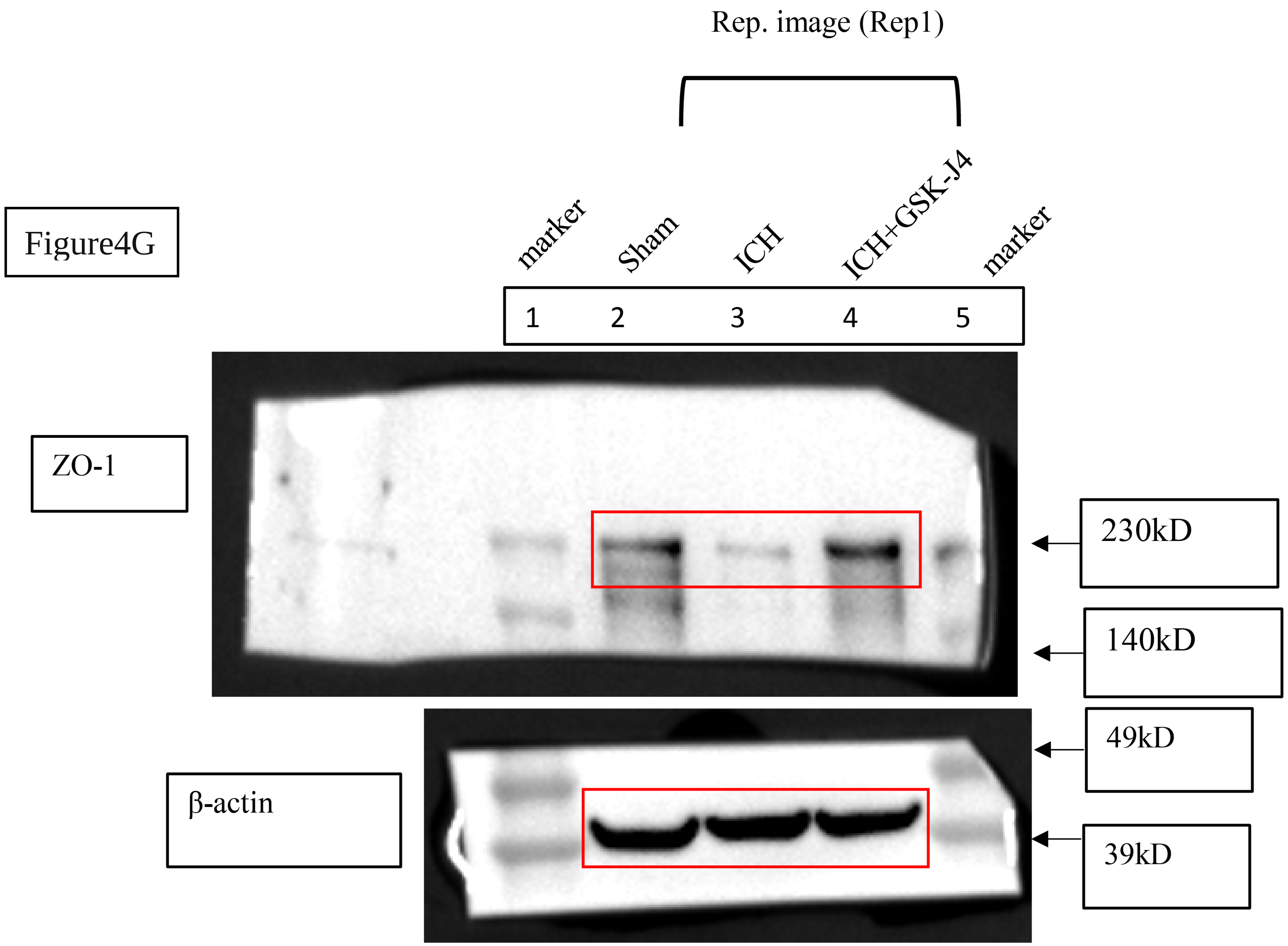
Figure4G
Rep. image (Rep1)
marker
Sham
ICH
ICH+GSK-J4
marker
1
2
3
4
5
ZO-1
230kD
140kD
β-actin
49kD
39kD

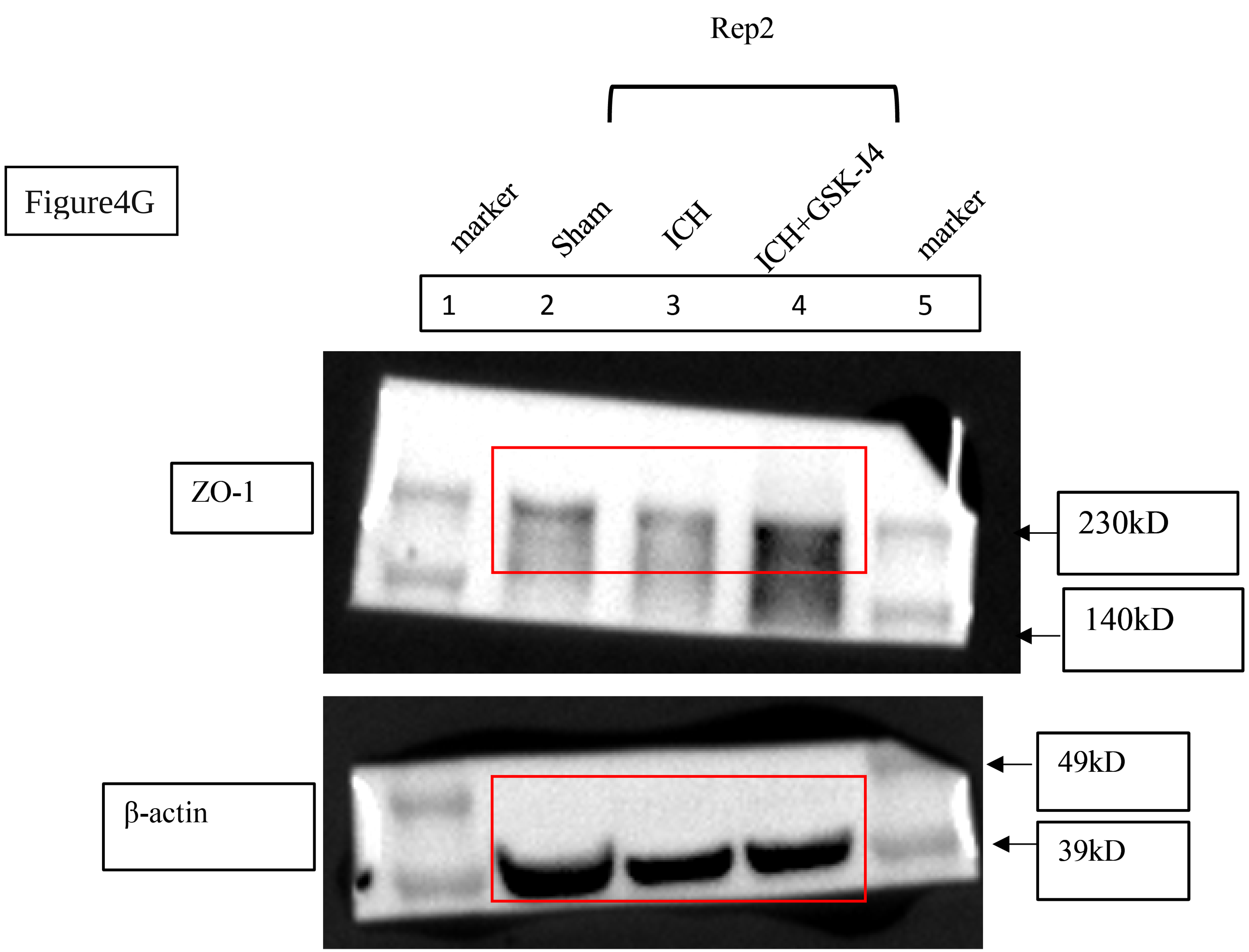
Figure4G
Rep2
marker
Sham
ICH
ICH+GSK-J4
marker
1
2
3
4
5
ZO-1
230kD
140kD
β-actin
49kD
39kD

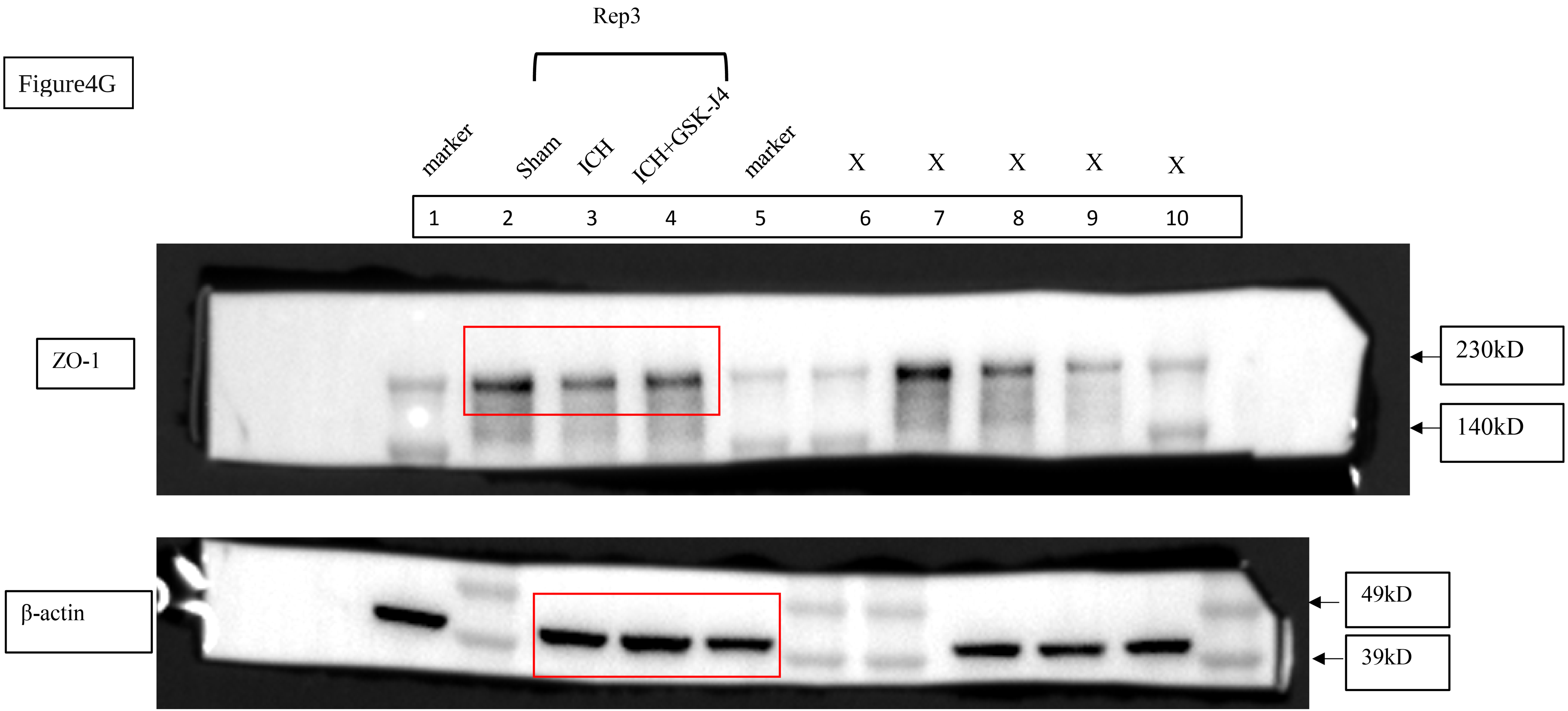
Figure4G
Rep3
marker
Sham
ICH
ICH+GSK-J4
marker
X
X
X
X
X
1
2
3
4
5
6
7
8
9
10
ZO-1
230kD
140kD
β-actin
49kD
39kD

Supplementary Figure S2. Original uncropped Western blot images corresponding to Figures 4A and 4G. Protein expression of iNOS, TNF-α, MMP-9, claudin-5, and ZO-1 in the perihematomal striatum of sham, ICH, and ICH + GSK-J4 mice was evaluated at 3 days post-ICH. For the ICH+GSK-J4 group, mice received intraperitoneal injections of 30 mg/kg GSK-J4 on days –3, –1, 1, and 3; ICH was induced on day 0. Red boxes indicate three independent replicates (Rep1–Rep3); the cropped areas from Rep1 were used in the main figures. Molecular weight markers are indicated on the right (in kDa). Unincluded lanes are marked with “X”.
