# Supplementary material for: Inhibition of Astrocytic JMJD3 Attenuates Neuroinflammation-Mediated Blood–Brain Barrier Disruption and Improves Functional Recovery After Intracerebral Hemorrhage in Mice
Source: Brain Sci. 2026 Apr 24;16(5):454. doi: 10.3390/brainsci16050454 (PMC13204954; doi:10.3390/brainsci16050454)
Supplement: Supplementary file 1 [file brainsci-16-00454-s001.zip › brainsci-4238353-supplementary/Supplementary Materials/Supplementary Figure S3.pdf]

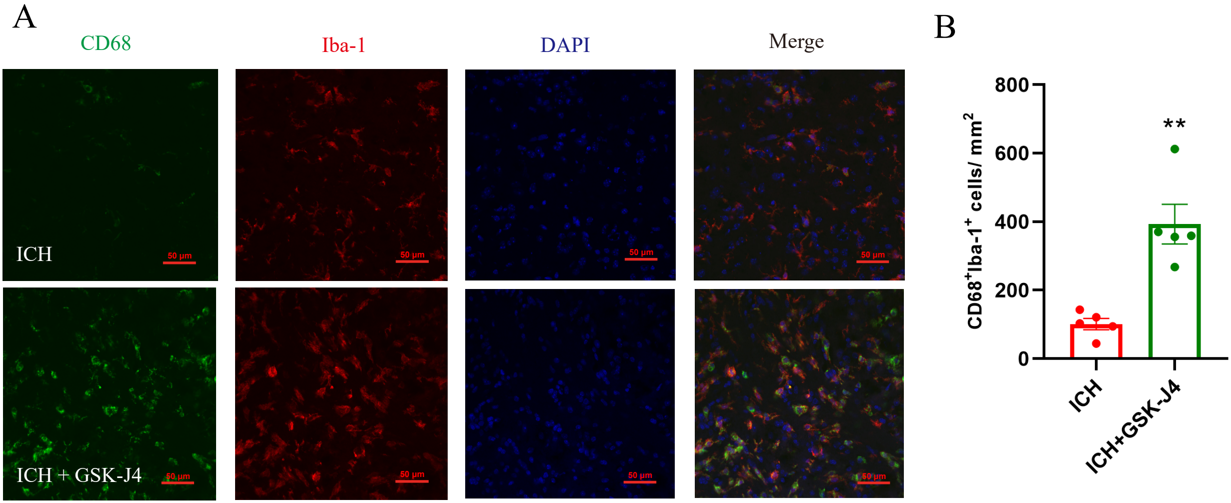


**Supplementary Figure S3.** Immunofluorescence staining of CD68 expression in Iba-1-postive microglia in ICH mice at 3 days post-induction, with or without GSK-J4 treatment. (A) Representative immunofluorescence images showing the co-staining of Iba-1 (microglia marker) and CD68 (phagocytic activity marker) in the perihematomal regions of ICH and ICH+GSK-J4 groups at 3 days post-ICH, indicating the effect of GSK-J4 on phagocytic microglia in acute ICH mice. Scale bars: 50 μm. (B) Quantitative analysis of the number of $CD68^{+}Iba\text{-}1^{+}$ microglia per $mm^2$, showing a significant increase of $CD68^{+}Iba\text{-}1^{+}$ double-positive microglia in ICH+GSK-J4 group, compared to ICH group. Values were averaged from three ROIs per mouse. Data are presented as mean ± SEM (n = 5 per group). $^{**}p < 0.01$ (two-tailed unpaired Student's t-test).
